# Genomic landscape and immune-related gene expression profiling of epithelial ovarian cancer after neoadjuvant chemotherapy

**DOI:** 10.1038/s41698-021-00247-3

**Published:** 2022-01-27

**Authors:** I. Lodewijk, A. Bernardini, C. Suárez-Cabrera, E. Bernal, R. Sánchez, J. L. Garcia, K. Rojas, L. Morales, S. Wang, X. Han, M. Dueñas, J. M. Paramio, L. Manso

**Affiliations:** 1 Biomedical Research Institute I+12, University Hospital “12 de Octubre”, Madrid, Spain; 2 Molecular Oncology Unit, Centro de Investigaciones Energéticas, Medioambientales y Tecnológicas (CIEMAT), Madrid, Spain; 3 Centro de Investigación Biomédica en Red Cáncer, Madrid, Spain; 4 Medical Oncology, University Hospital 12 De Octubre, Madrid, Spain; 5 Pathology Department, University Hospital 12 De Octubre, Madrid, Spain; 6 Medical Oncology, Vall d’Hebron University Hospital, Barcelona, Spain; 7 Experimental Medicine Unit, Oncology, GlaxoSmithKline, Waltham, MA USA; 8 Present Address: Medical Oncology, Infant Cristina University Hospital, Madrid, Spain

**Keywords:** Ovarian cancer, Ovarian cancer

## Abstract

Platinum-based neoadjuvant chemotherapy followed by interval debulking surgery is an accepted treatment for patients with stage III or IV epithelial ovarian cancer who are not suitable for primary debulking surgery. The identification of suitable adjuvant treatments in these patients is an unmet need. Here, we explore potential genomic characteristics (mutational and immune-associated expression profiles) in a series of patients undergoing neoadjuvant chemotherapy. Tumor samples from biopsy and interval debulking surgery were analyzed for mutational landscape and immune profiling, together with detailed immunohistochemistry using different immune cell markers, and correlated with clinicopathological characteristics and potential response to neoadjuvant chemotherapy. No major differences in the mutational landscape were observed in paired biopsy and surgery samples. Genomic loss of heterozygosity was found to be higher in patients with total/near-total tumor response. The immune gene expression profile after neoadjuvant chemotherapy revealed activation of several immune regulation-related pathways in patients with no/minimal or partial response. In parallel, neoadjuvant therapy caused a significant increase of tumor-infiltrating lymphocyte population abundance, primarily due to an augmentation of the CD8+ T cell population. Remarkably, these changes occurred irrespective of potential homologous recombination defects, such as those associated with BRCA1/2 mutations. Our study strengthens the use of loss of heterozygosity as a biomarker of homologous repair deficiency. The changes of immune states during neoadjuvant chemotherapy reveal the dynamic nature of tumor-host immune interactions and suggest the potential use of immune checkpoint inhibitors or their combination with poly-ADP polymerase inhibitors in high stage and grade epithelial ovarian cancer patients undergoing neoadjuvant therapy.

## Introduction

Ovarian cancer represents the seventh most commonly diagnosed cancer and the eighth leading cause of cancer-related death in women worldwide, with an estimated 295,414 new cases and 184,799 deaths annually^1^. Cancer of the ovary covers a histologically and genetically broad range of tumors, with high-grade serous ovarian cancer (HGSOC) being responsible for 70–80% of all ovarian cancer deaths^2^. HGSOC represents one of the subtypes of the most prevalent ovarian cancer type, epithelial ovarian cancer (EOC), which accounts for approximately 90% of the cases^3^. Endometrioid ovarian carcinoma (EOVC) represents an uncommon subtype of EOC, accounting for approximately 10% of all ovarian tumors^4^. The majority of patients with EOC present with advanced disease and disease management consists of platinum-based chemotherapy and cytoreductive surgery^5^. An alternative option is neoadjuvant chemotherapy (NACT) followed by interval debulking surgery (IDS), which has been shown to be safer and better tolerated than primary debulking surgery for patients with more advanced disease^5,6^. Despite initial chemosensitivity, most patients relapse, and ultimately platinum resistance ensues resulting in poor 5-year survival of 45%^7^. In the hope of emulating the success seen in other tumors, attention has turned to immunotherapy as a new strategy to improve survival in EOC^7^.

Understanding the complete genomic background of EOC will guide targeted therapies, which will pave the way for the use of precision medicine. Molecular characteristics such as *BRCA1/2* mutations and homologous repair deficiency (HRd) in EOC have been demonstrated and validated as predictive of response to platinum therapy and poly-ADP polymerase (PARP) inhibitors^8–10^. Given the molecular heterogeneity that exists within EOC, optimal patient care should be provided through *BRCA1/2* mutation status, mutation burden, homologous recombination (HR) deficiency, and tumor microenvironment (TME) immune composition. Another relevant factor in clinical outcome is intratumoral heterogeneity. This can be mediated, at least in part, by *TP53* mutations, which are extremely prevalent in EOC^11^ and drive chromosomal instability^12,13^. Moreover, intratumor heterogeneity together with HRd in EOC can be associated with prognosis and molecular subtype and, remarkably, may change in treatment course^14^.

Previous work has focused on the potential enhancement of the immune response by NACT in EOC^15–18^. Overall, NACT can have a significant impact on the TME by enhancing host immune response, including the increase of the CD8+ and T helper 1 (Th1) cell population as well as cytolytic tumor-infiltrating lymphocyte (TIL) population abundance, and the reduction of regulatory T (Treg) cell population^15^. This effect is tempered by high/increased levels of CTLA-4, programmed death-1 (PD-1), and programmed death-ligand 1 (PD-L1)^15^. However, these findings are still under discussion^16^. Sequential chemo-immunotherapy may improve disease control in EOC^15^. The most encouraging results in other tumor types have come from the blockade of the PD-L1/PD-1 axis. In EOC, activity in unselected patients has been modest with overall response rates of only 15%^19^. The addition of atezolizumab to a backbone comprised of bevacizumab and chemotherapy failed to significantly improve progression-free survival (PFS) in patients with newly diagnosed stage III/IV ovarian cancer, according to results from the phase 3 IMagyn050/GOG 3015/ENGOT-OV39^20^. This failure highlights the urgent need for novel immunotherapy strategies and validated immune biomarkers to guide patient selection. Immunotherapies may be more active in the context of small volume tumor burden and before immune exhaustion. Recent studies have started to define the interplay between mutational intratumor heterogeneity and T cell interactions^21^, as well as the potential effect of chemotherapy on T cell infiltration in HGSOC^15^. However, the extent of heterogeneity, its underlying mechanisms, and its impact on therapeutic response remain unknown^22^.

In this pilot study, we sought to identify clinical and molecular characteristics of prospectively collected metastatic peritoneal (omental) specimens before and after platinum-based NACT. We also attempted to identify genes or molecular pathways that might offer opportunities for personalized therapy for this subset of patients.

## Results

### Overview of the EOC cohort

From >600 patients with EOC entered in the Hospital 12 de Octubre Registry from 2000 to 2018, we selected a non-consecutive cohort of patients treated with NACT followed by IDS (Table 1). Associations of clinical data with response to NACT were explored. Most patients (75%) had advanced disease unresectable at presentation and received NACT with the goal of achieving cytoreduction. 83% of patients presented HGSOC. In most of the patients, we observed that CA125 antigen levels decreased after NACT and that this reduction is not related to response (Supplementary Fig. 1). Furthermore, around 40% of CRS3 patients (6/15) had not relapsed after a median follow-up time of 75.5 months (range 20–117 months) from IDS until the last follow-up date of this study.

**Table 1:** Characteristics of the patient and sample cohorts.

Characteristics	No. of patients	% Patients
Age (years)		
Median age at diagnosis	65	
Range	(41–84)	
Race or ethnic group		
Caucasian	57	96%
Latin	2	3%
Other	1	1%
BMI (kg/m^2^)		
Median	1.49	
Range	(1.25–1.89)	
Initial FIGO stage		
III	28	47%
IV	14	23%
Missing	18	30%
Histology		
High-grade serous	50	83%
High-grade endometrioid	2	3%
Missing	8	13%
NACT chemotherapy		
Every 3 weeks	44	73%
Weekly	9	15%
Other	7	12%
Interval debulking surgery		
Complete gross resection	35	58%
Incomplete resection	15	25%
Missing	10	17%
Radiological response		
Complete response (CR)	2	3%
Partial response (PR)	40	67%
Stable disease (SD)	1	2%
Missing	15	23%
Chemotherapy response score		
CRS1	11	18%
CRS2	18	30%
CRS3	16	27%
Unknown	15	23%
CA125 at diagnosis		
Mean (SD)	2010 (3134)	
Median (min, max)	978 (7.15936)	
CA125 post chemotherapy		
Mean (SD)	81 (112)	
Median (min, max)	27 (11.440)	
Relapse		
Yes	49	82%
No	11	18%
Median PFS (months)	14 (2.8–89)	

*BMI* body mass index, *NACT* neoadjuvant chemotherapy, *PFS* progression-free survival.

### Genomic features of EOC samples

The overall mean of alterations observed in our EOC samples was 3.75 and ranged from 1 to 14 alterations per sample. Numbers of non-synonymous short mutations, copy number alterations (CNAs), and rearrangements were similar between CRS1, CRS2, and CRS3 patient groups (Supplementary Table 1).

**Fig. 1:**
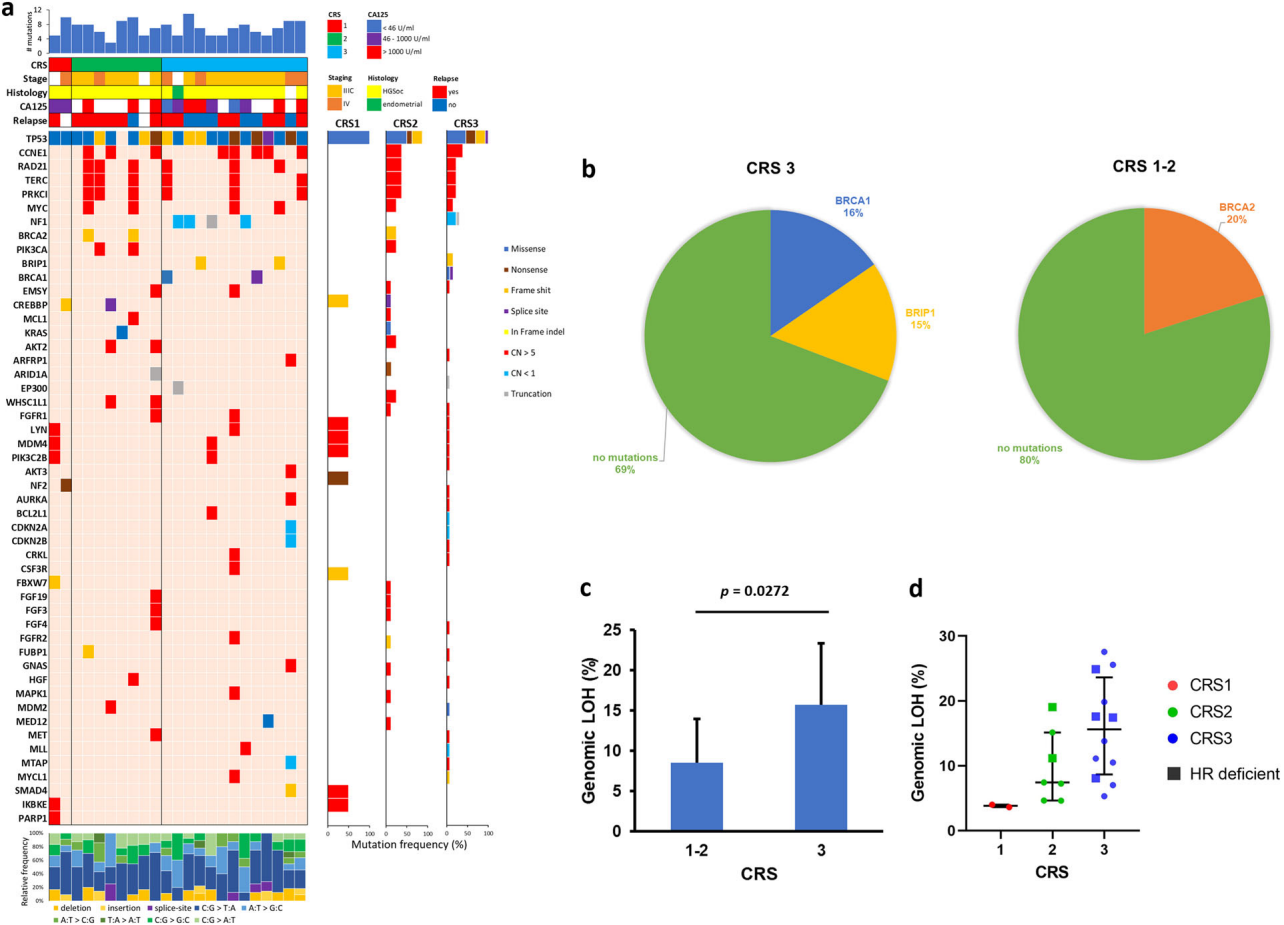
Genomic alterations in EOC biopsy samples. **a** Mutation plot showing individual EOC biopsy samples on the *x* axis from left to right ordered by type of response: CRS1, CRS2, and CRS3. Gene alterations are annotated according to the color panel on the right side of the image. The frequency of appearance of the mutation in the CRS group is plotted on the right panel. Mutation burdens and type of base-pair substitution and indels are displayed in the top and bottom panels, respectively. Clinical data are displayed in the top panel. **b** Frequency of CRS3 and CRS1/2 biopsy samples with HR pathway-related genomic alterations. **c** Average genomic LOH in CRS3 and CRS1/2 biopsy samples. **d** Genomic LOH in CRS1, CRS2, and CRS3 biopsy samples. Samples with alterations in HR pathway-related genes are indicated (HR deficient).

In biopsy samples (*n* = 23), we observed exonic alterations in 50 out of 309 genes (Fig. 1a). As expected, *TP53* mutations were detected in 22 of 23 patients in addition to frequent genomic CNAs^11^. Except for *TP53*, point mutations in other genes were not common. *NF1* was only found to be mutated in CRS3 in 31% of the samples (4/13). Apart from *NF1*, no associations were observed between the CRS subtype and mutations in tumor suppressor genes or oncogenes. Additionally, we did not observe differences in tumor mutational burden (TMB) (9.46 ± 4.46 for CRS1; 9.14 ± 2.41 for CRS2; 9.31 ± 2.33 for CRS3) or in the type of base-pair substitution and indels between patient groups (Fig. 1a).

We observed gene mutations related to the HR mechanism in *BRCA1*, *BRCA2,* and *BRIP1*. HR gene mutations were found to be slightly more frequent in NACT responder patients (CRS3) than in non/partial-responders (CRS1/2) (Fig. 1b) but did not reach statistical significance.

We also evaluated genome-wide loss of heterozygosity (LOH) as a potential indicator of HRd. We observed that tumors from responder patients to NACT (CRS3) showed higher genomic LOH than tumors from non/partial-responders (CRS1/2) (Fig. 1c). Remarkably, samples with mutations in HR genes and a higher LOH are almost all derived from responder patients in our cohort (Fig. 1d).

### Tumor-specific immune features

Using nCounter gene expression data, we evaluated the immune profile of EOC before treatment with NACT. After quality control and normalization, 25 of the 32 biopsy samples were included in this evaluation.

We did not observe any significant difference in either the immune gene signature or immune cell infiltration between EOC biopsy samples from patients with no or minimal tumor response (CRS1; *n* = 3), partial response (CRS2; *n* = 9), and total/near-total tumor response (CRS3; *n* = 13) to NACT (Supplementary Figs. 2 and 3).

We next examined immune gene expression as well as immune cell infiltration in the biopsy samples from HR-deficient tumors and HR-proficient tumors. We did not observe significant differences in immune profile between HR-deficient and proficient biopsy samples or between stage IIIC and stage IV diagnosed biopsies (data not shown).

### Genomic analysis of paired biopsy and surgery lesions

**Fig. 2:**
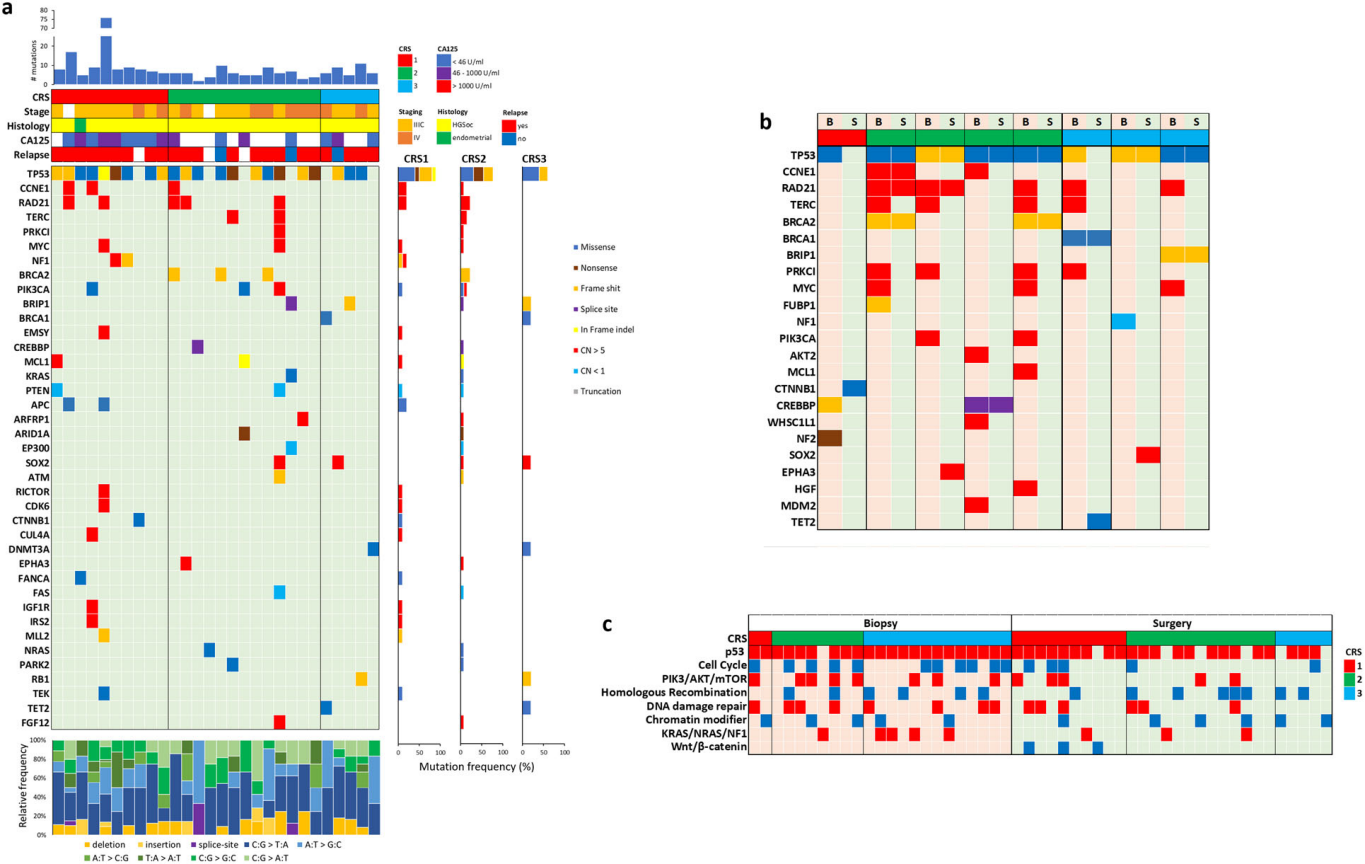
Genomic alterations in EOC surgery samples. **a** Mutation plot showing individual EOC surgery samples on the *x* axis from left to right ordered by type of response: CRS1, CRS2, and CRS3. Gene alterations are annotated according to the color panel on the right side of the image. The frequency of appearance of the mutation in the CRS group is plotted on the right panel. Mutation burdens and type of base-pair substitution and indels are displayed in the top and bottom panels, respectively. Clinical data are displayed in the top panel. **b** Mutation plot of paired biopsy–surgery samples. B biopsy, S surgery. Gene alterations are annotated according to the color panel on the left side of the image. **c** Alterations in biological process and pathways in biopsy and surgery samples. Red and blue squares indicate a sample with at least an alteration in the indicated pathway.

In surgery samples obtained at IDS after NACT (*n* = 28), 39 out of 309 genes were found to be altered (Fig. 2a). As in our biopsy samples, *TP53* represented the most frequently mutated gene (22/28) and CNAs were very prevalent. No differences in TMB (21.94 ± 35.51 for CRS1; 7.37 ± 2.76 for CRS2; 9.58 ± 3.03 for CRS3) were observed between patient groups.

We studied genomic changes in the tumor at IDS after NACT in paired biopsy-surgery samples (Fig. 2b). After quality control, 8 of the 11 paired biopsy-surgery samples were included in this evaluation. We observed loss of *MYC* amplification in three surgery samples (CRS2; *n* = 2 and CRS3; *n* = 1), two of which additionally showed loss of *RAD21* gene amplifications. It is of note that the *RAD21* gene is located downstream of *MYC* on chr8 q24. Additionally, loss of amplification in PI3K pathway genes was observed in surgery samples from three CRS2 patients, two of whom showed loss of *PIK3CA*, whereas another displayed loss of *AKT2* amplification. Additionally, four tumors (CRS2; *n* = 3 and CRS3; *n* = 1) showed loss of amplification in *TERC* and *PRKC1*, which are located downstream of *PIK3CA* on chr3 q26. In contrast, we did not see the gain or loss of mutations in HR-related genes after NACT. Amplification count revealed that NACT promoted the loss of amplification, which does not seem to be associated with the type of response to chemotherapy, although it may indicate a partial reduction in tumor cell load in surgery samples (Supplementary Fig. 4). In line with this, we found that LOH scores greatly decreased after NACT in 4 out of 5 paired samples (Supplementary Fig. 5). No relevant changes in short mutations were observed in our paired samples after NACT.

Alterations in different genes may contribute to the functionality of biological processes or pathways. For instance, we found more mutations in the KRAS/NRAS pathway in biopsy samples from total/near-total responders to NACT (CRS3; *n* = 13) than in biopsy samples from no/minimal responder patients (CRS1; *n* = 2) or partial responders (CRS2; *n* = 8) (Fig. 2c). On the other hand, more mutations in the PI3K pathway were observed in tumors from CRS1 and CRS2 patients compared to tumors from CRS3 patients. Contrarily, the WNT/beta-catenin pathway is only altered in three surgery samples from no/minimal responder patients. Moreover, we found that some pathways might be mutually exclusive in total/near-total responders to NACT (Supplementary Table 2).

### Analysis of immune profile change by NACT

We hypothesized that carboplatin/paclitaxel-based NACT might provoke a beneficial immune profile in EOC lesions for subsequent treatment with immunotherapy. nCounter gene expression analysis showed a significant stimulation for several pathways related to immune regulation after NACT treatment in the CRS1/2 patient population, including immune cell adhesion and migration (*p* = 0.0120), lymphoid compartment (*p* = 0.0270), and myeloid compartment (*p* = 0.0041), whereas no significant stimulation of those pathways was observed in the CRS3 patient population (Supplementary Fig. 6). Immunosuppressive transforming growth factor (TGF)-beta signaling was found to be significantly stimulated after NACT in CRS3 patients (*p* = 0.0013). These results suggest that CRS3 patients might not benefit from maintenance treatment with immunotherapy after the initial response to NACT.

**Fig. 3:**
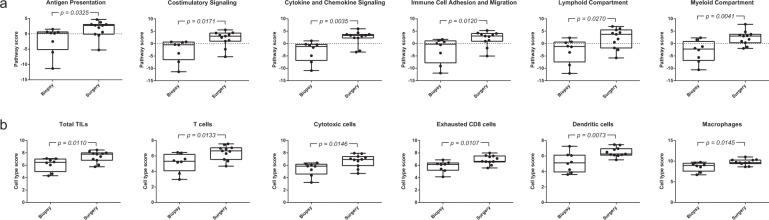
Increase of inflammatory signature in EOC surgery samples from patients with no/partial response to NACT. **a** Several pathways associated with immune regulation are stimulated by NACT, including antigen presentation, costimulatory signaling, cytokine and chemokine signaling, immune cell adhesion and migration, lymphoid compartment, and myeloid compartment. Pathway scores were used to summarize data from a pathway’s genes into a single score. **b** Different immune cell subsets show increased abundance in surgery samples after NACT, including total TILs, T cells, cytotoxic cells, exhausted CD8 cells, dendritic cells, and macrophages. Cell-type scores were calculated as the average log2 normalized expression of each cell’s marker genes. As cell-type scores are calculated in log2 scale, an increase of 1 on the vertical axis corresponds to a doubling in abundance. The value for each EOC sample is shown, biopsy samples *n* = 8 and surgery samples *n* = 11. *p* Values were determined using the unpaired *t* test for samples with a Gaussian distribution and Mann–Whitney test for samples without normal distribution.

Next, nCounter gene expression analysis was used for the further immunological analysis of EOC lesions from CRS1/2 patients before and after treatment with NACT. From the 19 paired biopsy-surgery samples available, 12 matched samples were obtained from CRS1/2 patients whereas the other 7 paired samples were derived from the CRS3 patient group. After quality control and normalization, we included 8 biopsy samples and 11 surgery samples from the 12 paired biopsy-surgery samples collected from CRS1/2 patients. We observed an increase of the inflammatory signature after NACT when evaluating biopsy and surgery samples from patients with no/partial response. Several immune-related genes showed enhanced expression in surgical samples compared to biopsy samples including *CCL14*, *NFKBIA*, and *IL33* as well as *S100A12*, *FOSL1*, *IL6*, and *STAT4* (Supplementary Fig. 7). Additionally, a significant stimulation after NACT treatment has been found for several pathways related to immune regulation including antigen presentation (*p* = 0.0325), co-stimulatory signaling (*p* = 0.0171), cytokine and chemokine signaling (*p* = 0.0035), immune cell adhesion and migration (*p* = 0.0120), lymphoid compartment (*p* = 0.0270) and myeloid compartment (*p* = 0.0041) (Fig. 3a), whereas immunosuppressive TGF-beta signaling does not show significant changes (data not shown).

**Fig. 4:**
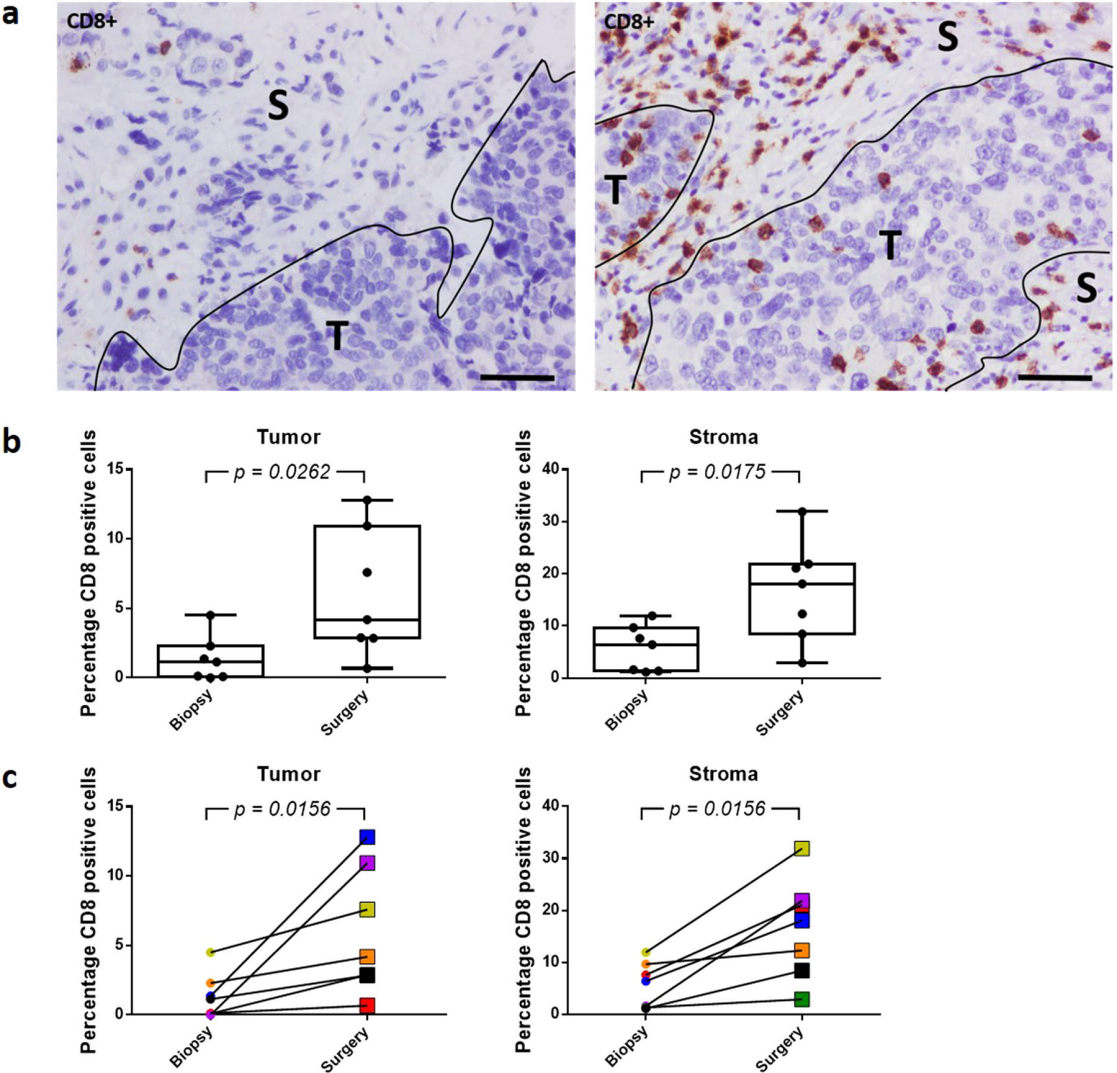
NACT enhances CD8+ cell infiltration in both tumor and stromal areas of the EOC sample. **a** Immunohistochemistry showing the presence of CD8+ cells in the EOC sample before (left) and after (right) NACT. S stroma, T tumor. Scale bars, 100 μm. **b** Plot of the percentage of CD8+ cells in the tumor and stromal areas before (biopsy) and after (surgery) NACT. The value for each EOC sample is shown. *p* Values were determined using Mann–Whitney test. **c** Line graph representing the percentage of CD8+ cells in the tumor and stromal areas in paired biopsy-surgery samples. Each line represents an EOC patient, with colors defining the same patient in both analyses. *p* Values were determined using the Wilcoxon test.

In agreement with the abovementioned data, several immune cell scores were significantly higher in EOC lesions at IDS after NACT treatment, indicating increased immune cell population abundance (Fig. 3b). NACT caused a significant 1.7-fold increase of the TIL population abundance (*p* = 0.0110). We also observed that the elevation of the T cell population (1.3 units; 1.7-fold difference) after NACT is primarily due to an augmentation of the CD8+ T cell population (*p* = 0.0144). The CD8+ T cell population showed a 1.3 unit increase, equivalent to a 1.7-fold difference, in surgical samples compared to biopsy samples, whereas the Th1 cell population did not show a significant difference (Supplementary Fig. 8). Immunohistochemistry analysis of the samples included in the nCounter gene expression analysis confirmed these observations, showing a significantly increased infiltration of the CD8+ T cell population in both tumor and stromal areas (*p* = 0.0262 and *p* = 0.0175, respectively) (Fig. 4a, b). Evaluation of this CD8+ T cell population revealed a 1.3 unit enhancement of the cytotoxic cell population and 1.0 unit augmentation of the exhausted CD8 cell population, corresponding to a, respectively, 1.7- and 1-fold increase in cell population abundance (Fig. 3b).

Additionally, we observed that the average dendritic cell, macrophage, neutrophil, and mast cell scores were, respectively, 1.3, 1.1, 2.1, and 1.9 units higher in EOC lesions after NACT treatment, indicating a 1.7-fold increase in dendritic cells (*p* = 0.0073), 1.2-fold increase in macrophages (*p* = 0.0145), 4.4-fold increase in neutrophils (*p* = 0.0002) and 3.6-fold increase in mast cell (*p* = 0.0037) population abundance (Fig. 3b and Supplementary Fig. 8). The natural killer (NK) cell population, Th1 cell population, and Treg cell population only showed a slight enhancement that did not reach statistical significance (data not shown). However, the steep decrease of Th1 cell: TIL population and Treg cell: TIL population ratios when comparing surgical samples with biopsy samples (*p* = 0.0325 and *p* = 0.0125, respectively) further emphasizes the augmentation of the TIL population after NACT (Supplementary Fig. 9).

The elevated immune cell population abundance provoked by NACT and observed in IDS samples was verified by the analysis of biopsy and surgery samples of each patient separately. In this analysis, only CRS1/2 patients with paired biopsy-surgery samples available after quality control and normalization were included (8 of the 19 paired cases). Except for the T cell population, a significant change in cell presence at the individual level was observed for all the abovementioned significantly increased immune cell types (Supplementary Fig. 10). Moreover, immunohistochemistry analysis of those samples included in the nCounter gene expression analysis showed a significantly enhanced infiltration of the CD8+ T cell population in both the tumor and stromal areas (*p* = 0.0156) for each individual (Fig. 4c).

**Fig. 5:**
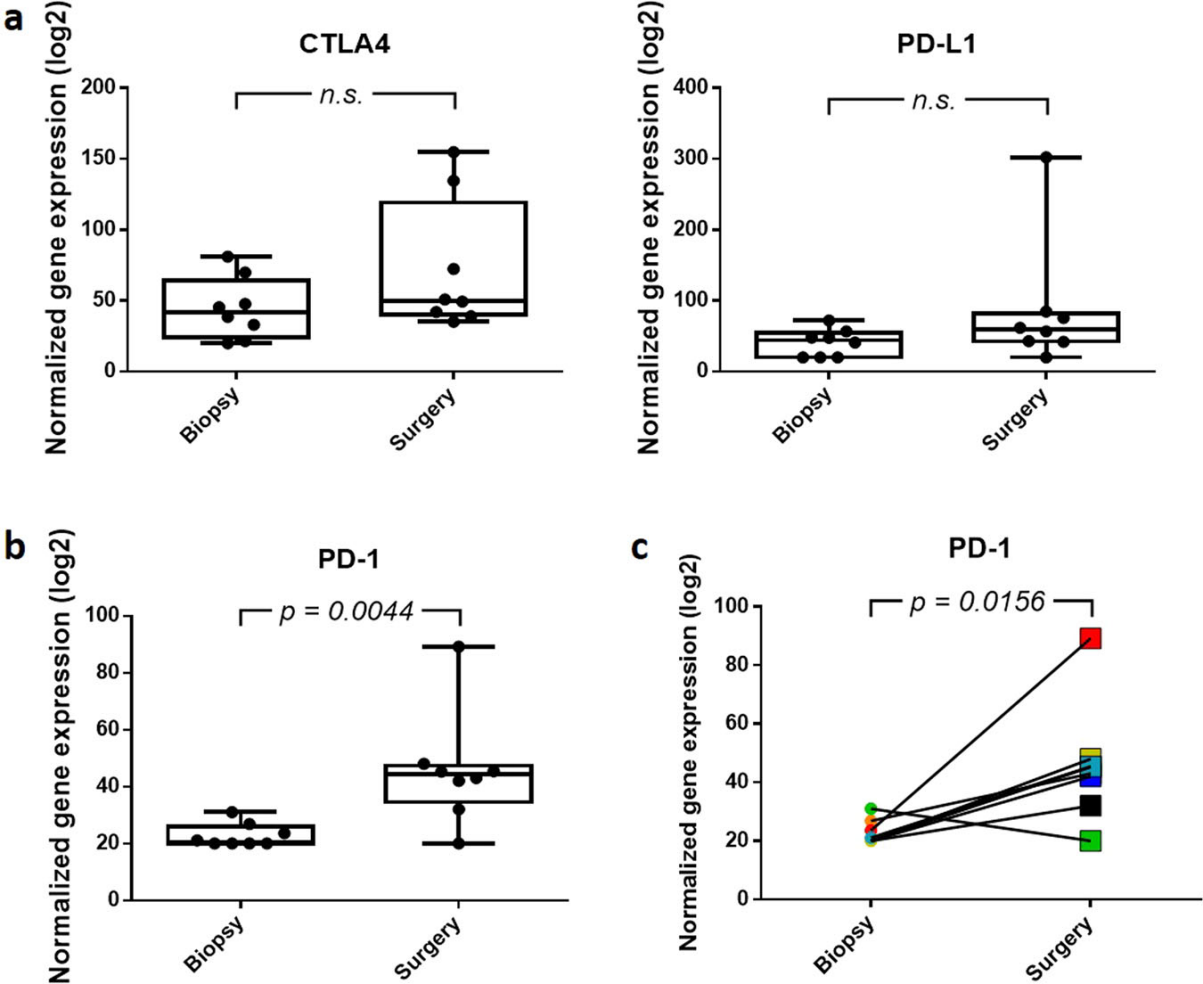
Normalized gene expression of *CTLA4*, *CD274* (PD-L1), and *PDCD1* (PD-1) before and after NACT. **a** No significant differences were observed in the CTLA4 and PD-L1 expression between biopsy and surgery EOC samples. The value for each EOC sample is shown. n.s. *p* > 0.05. **b**, **c** NACT causes a significant increase in PD-1 expression. The plot of PD-1 expression in which the value for each EOC sample is shown (**b**). *p* Value was determined using Mann–Whitney test. Line graph representing the increase in PD-1 expression in paired biopsy–surgery samples (**c**). Each line represents an EOC patient. *p* Values were determined using the Wilcoxon test.

Finally, we evaluated the expression of immune checkpoint proteins CTLA-4, PD-1, and PD-L1 in the abovementioned patient population with paired biopsy-surgery samples and CRS1/2. Even though *CTLA4* and *CD274* expression only showed a slight tendency towards enhanced expression, a significant increase in the *PDCD1* expression level was observed in surgery samples compared to biopsy samples, both in general (*p* = 0.0044) and at the individual level (*p* = 0.0156) (Fig. 5).

### Correlation of mutational characteristics with increased immune cell infiltration

**Fig. 6:**
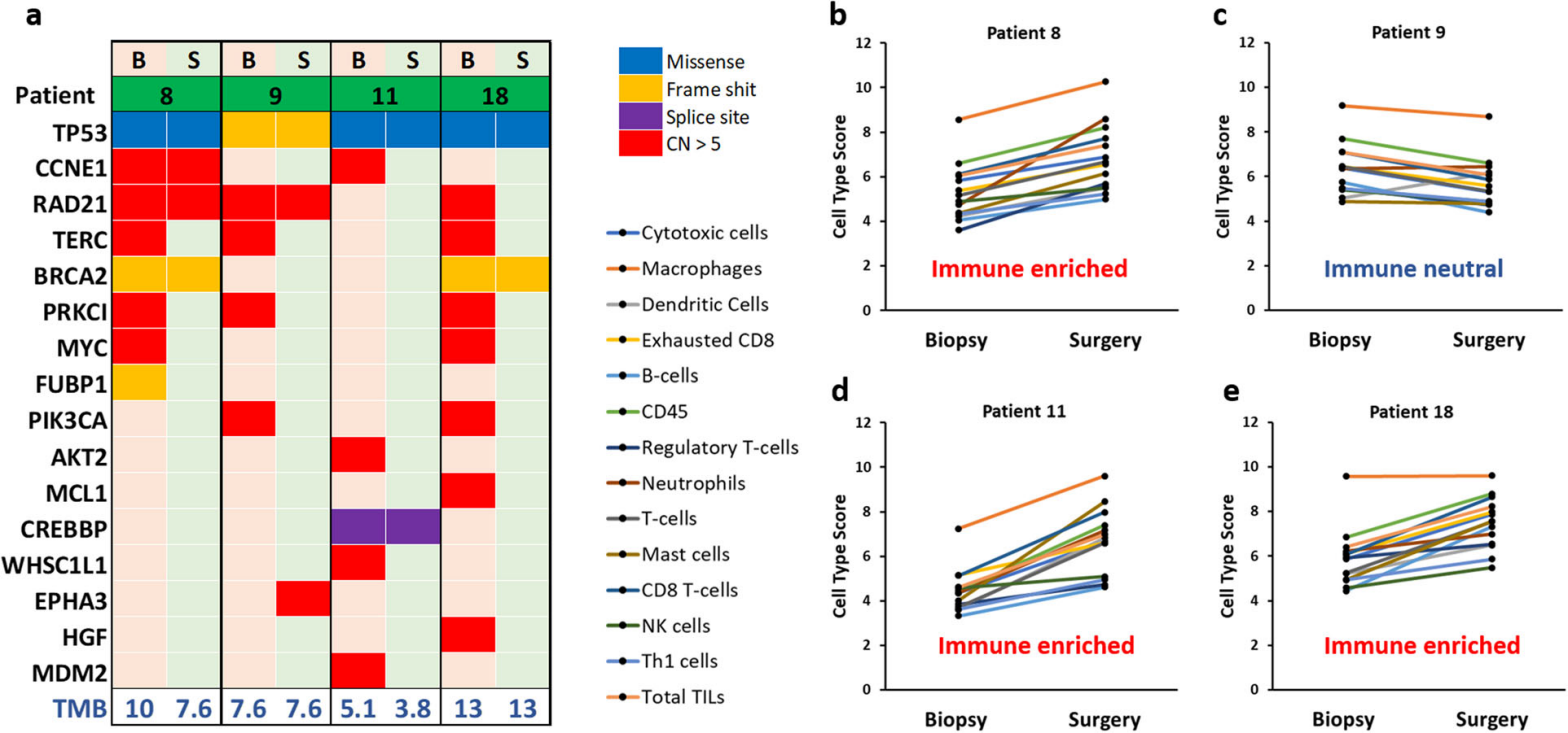
Correlation of genomic alterations with increased immune cell infiltration in EOC surgery samples. **a** Mutation plot of paired biopsy–surgery samples that coincided with paired biopsy–surgery samples used for immune profiling by nCounter gene expression analysis. B biopsy, S surgery. Gene alterations are annotated according to the color panel at the left side of the image. **b**–**e** Line graphs representing the abundance of immune cell populations in paired biopsy–surgery samples of patients 8 (**b**), 9 (**c**), 11 (**d**), and 18 (**e**) before and after NACT. TMB tumor mutational burden.

We ultimately tried to correlate genomic features with the increased immune cell scores found in the CRS1/2 population. From the 8 paired biopsy-surgery samples that were subjected to nCounter gene expression analysis, only 4 paired samples coincided with the paired samples included in the comprehensive genomic profiling analysis. We divided those paired samples in two groups: 1) samples that showed a high increase in the average immune cell infiltration score after NACT (so-called “immune enriched”), and 2) samples that did not show or only showed a small increase in the average immune cell infiltration score (so-called “immune neutral”). Of the 4 paired biopsy-surgery samples (all derived from CRS2 patients), analyzed by both comprehensive genomic profiling and nCounter gene expression, 3 were classified as “immune enriched” whereas the other paired sample was listed as “immune neutral”. We observed loss of amplification of *MYC* and *MDM2* in the “immune enriched” samples, whereas amplification of other genes was found in the “immune neutral” sample (Fig. 6).

## Discussion

The current clinical management of EOC often includes NACT in order to decrease tumor burden prior to IDS. Besides allowing tumor debulking which facilitates posterior surgeries, NACT may facilitate further treatments. In this regard, previous studies have focused on possible modulation of the immune environment by NACT^15–18^. However, there is some controversy regarding whether NACT may facilitate immunotherapy, as suggested by increased infiltration of CD8 CTLs^15–18^ and B cells^17^ as well as high stromal CD8+/FOXP3 and CD3+/FOXP3+ ratio after NACT^17^. Our present data may reinforce these findings. In addition, our studies indicate that such potential immune enhancement is related to the potential response to NACT displayed by the patients and provides some explanation to the previous controversies^15–18^. In this regard, our data might suggest that patients showing poor responses to NACT are good candidates for immunotherapies. Nonetheless, most previous studies do not include genomic characterization of the tumors, with the clear exception of the immunogenomic studies by Jimenez et al. in multi-peritoneal baseline tumors of HGSOC^22^, suggesting that NACT might induce immune activation with increased NK cell and T cell infiltrations, but restricted to specific locations due to possible tumor heterogeneity^22^.

The use of initial biopsy and IDS provides the opportunity to collect tumor samples before and after a defined treatment intervention to study the molecular profile of the tumor and identify potential biomarkers, which may guide the development of novel targeted therapies. In this regard, beyond *BRCA1/2* mutation status, no other biomarker in EOC enables up-front and precise identification of patients with platinum-sensitive or platinum-resistant disease. Moreover, besides the potentially difficult identification of these biomarkers, it is important to consider that tumor heterogeneity may affect clinical outcomes due to the emergence of minority clones that could present resistance to these therapies.

In addition to this intrinsic tumor-specific heterogeneity, it is important to consider tumor variation during disease progression or during therapy that can also affect the response. For example, the presence and functionality of immune cells in the TME may affect the effectiveness of different antitumor therapies, including immunotherapy. In this context, we have analyzed possible changes in the genomic alterations and changes in immune cells and their functionality during the NACT of EOC. The presence or absence of intratumoral T cells correlates with the clinical outcome of advanced ovarian carcinoma after surgical debulking and adjuvant chemotherapy^23^. NACT can have a significant impact on the TME, with more than half of EOCs showing increased lymphocytic infiltration and upregulation of PD-L1 expression post-chemotherapy^24^. Our study showed an increase in the inflammatory signature in surgery samples from CRS1/2 patients after NACT. Several pathways associated with immune regulation have been found to be stimulated and different immune cell subsets show increased abundance. Not only was an increase of functional cells found, but also an increase of exhausted cells. Nevertheless, the elevation in the functional immune cell population is higher than the increase in the exhausted population. Infiltration of the CD8+ T cell population has been observed not only in the stroma beyond the tumor but also in the tumor itself, thus emphasizing the possible effect of immunotherapy. Nevertheless, we did not observe any significant differences in the immune gene signature or immune cell infiltration between initial biopsy samples from the different patient groups.

The study of mutational characteristics correlated with a high increase of immune cell populations after NACT revealed that 2 out of 3 paired samples showed loss of *MYC* amplification, whereas the additional paired sample showed loss of *MDM2* amplification. As *MYC* amplifications have been associated with immune exclusion in tumors^22^, the observed increase of immune cells could be explained by the reduction of *MYC* amplifications after NACT. Besides, Zou et al. published that MDM2 can act as an E3 ubiquitin ligase and reduce T cell activation by degradation of transcription factor NFATc2, which results in the resistance to PD-1 inhibitors. Therefore, the loss of *MDM2* amplification after NACT may allow immune activation^25^.

We further found that the disease stage at diagnosis might be correlated with response to NACT. This association seems to be rather based on the genomic profile of the tumor, as no differences were observed between biopsy samples from patients with diagnosed Stage IIIC and diagnosed Stage IV disease. Gene alterations observed in our biopsy cohort showed a high intertumor heterogeneity, characteristic of EOC^26^ and similar to surgery samples from TCGA Ovarian PanCancer Atlas, especially in amplification (Supplementary Data 1). *TP53* and *NF1* were the most frequently mutated genes in our cohort as well as in samples from TCGA. Although the frequencies of samples with *BRCA1* mutations [2/23 = 8.7% in biopsy samples (all CRS3) and 2/28 = 7.1% in surgery samples (one CRS1 and one CRS3)], *BRCA2* mutations [2/23 = 8.7% in biopsy samples (all CRS2) and 3/28 = 10.7% in surgery samples (all CRS2)] and *BRIP1* mutations [2/23 = 8.7% in biopsy samples (all CRS3) and 2/28 = 7.1% in surgery samples (one CRS2 and one CRS3)] in our cohort were slightly higher than in samples from TCGA (*BRCA1* = 3.4%, *BRCA2* = 2.9%, *BRIP1* = 1.0%) (Supplementary Data 1) and we did not detect mutations in other HR-related genes (*ATM*, *BARD1*, *CHEK2*, *NBN*, *PALB2*, and *RAD51D*), the differences in percentage of samples with gene mutations could be due to different size of cohorts. TCGA Ovarian PanCancer Atlas includes 585 samples whereas our cohort, analyzed by comprehensive genomic profiling, existed of 23 biopsy and 28 surgery samples. The lower number of samples in our cohort could provoke that small changes in mutation number produce bigger changes in alteration frequencies. Additionally, frequencies of short mutations in *ATM*, *BARD1*, *CHEK2*, *NBN*, *PALB2*, and *RAD51D* were found to be all <2% (1 per 50 samples) in TCGA Ovarian PanCancer, which could explain the absence of detected alterations in those genes in our cohort. The limited size may also determine why our analyses failed to describe a potential genomic biomarker that may predict NACT response in biopsies.

When HR is impaired (HRd), often as a result of genetic changes in the key players, less-precise forms of DNA repair are used, such as non-homologous end joining. This results in the induction of point mutations or frequent deletions and has been implicated in the progression of a range of cancers^27^. Consequently in HR-deficient tumors, activated DNA repair mechanisms are less precise and may cause frequent deletions, potentially leading to better responses to chemotherapy^28^. In our cohort, we observed a non-significant increased representation of alterations in HR genes in responders to NACT. However, quantifying the genomic LOH provides the possibility of measuring HRd as a biomarker^29^. The extent of genome-wide LOH may identify BRCA wild-type patients who have other alterations not recognizable by the assessment of short mutations in HR pathway genes and could provide a single global assessment of HRd irrespective of the causative lesion, and the potential for using it as a therapeutic target. This aspect is of particular relevance as these tumors usually possess sensitivity to PARP inhibitors^30^. In our series, we observed that responders to NACT had a significantly higher genomic LOH than non-responders which is in agreement with previous reports^14^.

In addition to a beneficial profile for treatment with platinum-based chemotherapy and PARP inhibitors, HR-deficient tumors have been associated with possible responses to immunotherapy^31–33^. However, HRd in our biopsy samples was not related to differences in the immune profile of the tumor compared to HR-proficient tumors. These results, which might be explained by intrinsic molecular subtype and/or the absence of significant differences in the TMB of biopsy samples, indicate that analysis of the immune profile of biopsy samples from EOC patients does not allow a proper stratification of patients that will respond to NACT or might benefit from immunotherapy.

Identified changes in intratumor heterogeneity during NACT in EOC are still limited. Takaya et al. recently reported that chromosomal instability, frequently associated with *TP53* mutations, decreased after NACT, leading to the appearance of chemoresistant clones^14^. We also observed loss of these *TP53* alterations in two out of eight paired samples, in spite of the fact that the *TP53* alterations showed similar prevalence before and after NACT. Additionally, we found that LOH scores greatly decreased after NACT in four out of five paired samples, which coincides with previous reports^14^. Our analyses further showed that no major genomic alterations occurred in tumors as a consequence of NACT, as we only identified two novel mutations, which affect *CTNNB1* and *TERT*, in surgery compared to biopsy samples.

The improvement in the outcome of patients with EOC is a currently unmet clinical need, as, despite an initial response to NACT and surgery, tumors recur. Immunotherapies have recently gained considerable attention in this field. However, as in many other tumor types, the challenge remains to determine what treatment combinations are most suitable and which patients are likely to benefit. In addition, it is important to identify how immunotherapy should be incorporated into current EOC management^34^. Our data showing deep changes in immune compartments after NACT could be of extreme relevance in this context. Our observations that immune states undergo changes during NACT treatment underscore the dynamic nature of tumor-host immune interactions. Immune responses to chemotherapies appear to be strongly influenced by pre-existing immune features in untreated tumors^35^, indicating that tumor immunogenicity is largely an intrinsic property. This may provide a potential explanation for the good prognosis of immune infiltrate molecular subtypes of EOC. Our data indicate that immune dynamics change over time during chemotherapy and, accordingly, monitoring longitudinal changes is required in order to make an accurate prediction of therapeutic response and to design possible new lines of therapy with immune checkpoint inhibitors (ICIs). In this regard, the presence of specific gene signatures and increased presence of effector immune cells, together with an increased expression of PD-1, point to the suitability of ICI use after NACT and surgery in EOC. On the other hand, the analysis of immune compartments of cells in biopsies might not be of sufficient validity to consider patients as candidates for ICI treatments.

Based on the results of this study, the CRS1/2 patient population that shows no or only partial response to NACT might be beneficial candidates for the treatment with ICIs. In those patients, NACT has been found to provoke a beneficial immune profile for subsequent treatment with immunotherapy. Nevertheless, other factors such as changes in the TMB as well as mutation-derived antigens are known targets of immunotherapy and their influence in the response to ICIs should be taken into account. As previously mentioned, we did not find any significant alterations in the TMB between biopsy samples from CRS1/2 and CRS3 patients. Of note, the lack of differences in the TMB might be due to the limited number of samples or to a real absence of difference in TMB after NACT. Although TMB was typically proposed as a predictive biomarker of response to ICI therapy, contradictory data was published^36^. Furthermore, the predictive value of TMB has been found to be tumor-dependent and ovarian tumors are classified among those with the lowest correlation between TMB and T cell–inflamed gene expression profile^36^. It is, therefore, plausible that TMB may be an indicator of genomic instability since ovarian cancer is characterized by mutations in DNA damage repair mechanisms, rather than a predictor of response to ICI. The change in TME in CRS1/2 patients, however, has been clearly demonstrated with a marked increase in immune cell infiltration and an increased expression of PD-1. Even though this TME might indicate the potential efficacy of immunotherapy in this patient population, specific targets for ICIs remain to be identified.

Collectively, our study provides an important insight into the evolution and mutational processes occurring in EOC, and how these are being affected by NACT. One clear limitation of our study is the limited number of samples analyzed, mostly in paired biopsy-surgery pairs, which needs further confirmation in larger longitudinal series. Nonetheless, even though the restricted study, our data can pave the way to new therapeutic avenues in the management of EOC after NACT.

## Methods

### Sample inclusion criteria

We identified patients from the Hospital 12 de Octubre Registry diagnosed with EOC who underwent NACT followed by IDS. We selected patients who: (1) had advanced high-grade EOC stage III-IV according to the FIGO classification; (2) received NACT with carboplatin and paclitaxel (weekly or three-week schedule based on patient characteristics); and (3) had EOC samples collected from primary lesions before patients underwent carboplatin/paclitaxel-based NACT (so-called “biopsy samples”), and from residual (matched) lesions at IDS after treatment with chemotherapy (so-called “surgery samples”) according to availability. Response to therapy was scored following guidelines recommended by the European Society for Medical Oncology and European Society of Gynaecological Oncology, using the chemotherapy response score (CRS)^5^. This score stratifies patients with no/minimal tumor response (CRS1), partial response (CRS2), and total/near-total tumor response (CRS3) to NACT.

**Fig. 7:**
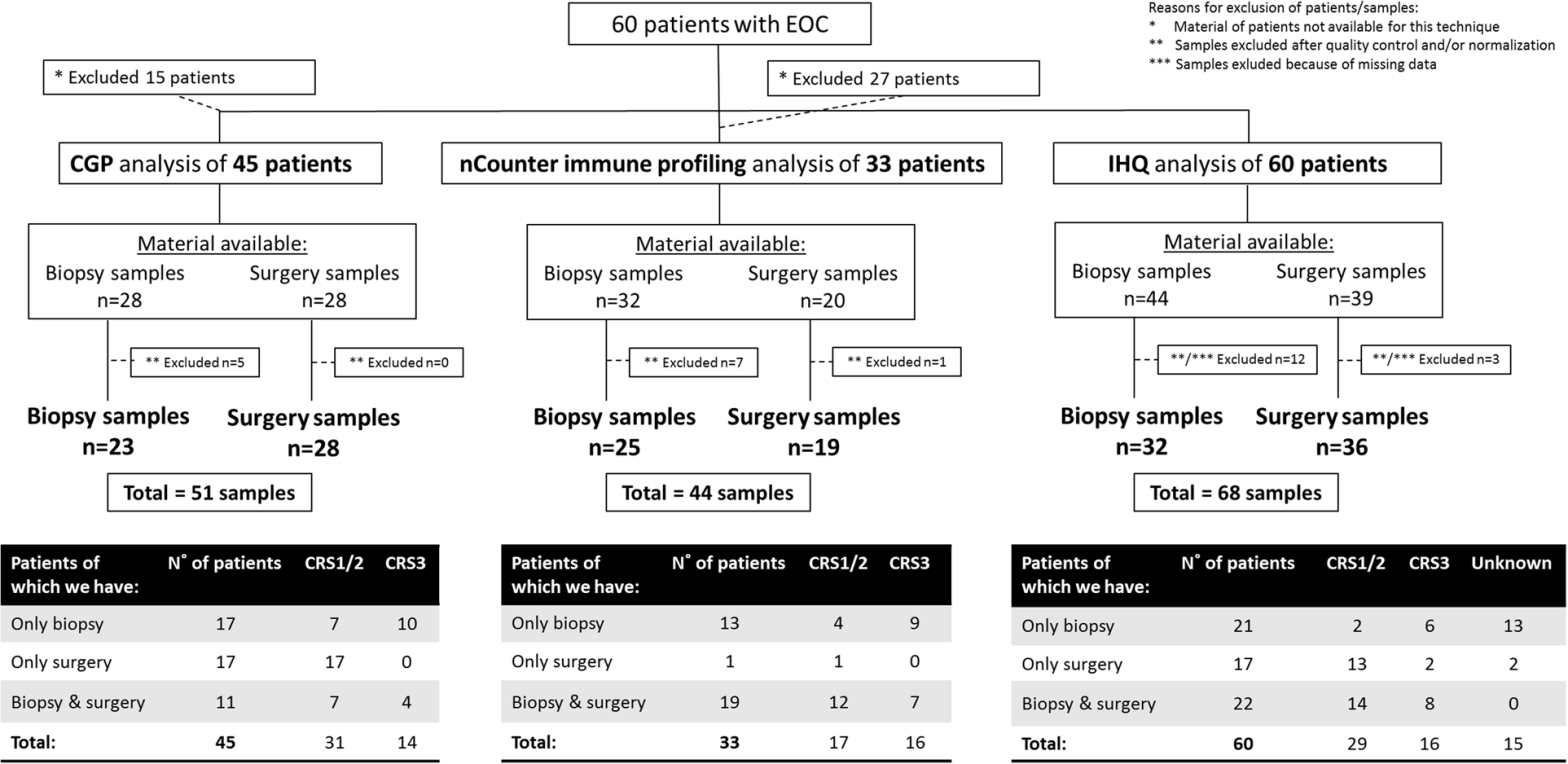
Scheme of study design detailing ovarian cancer patient cohort and material available for this study. Samples from 60 patients with stage III–IV epithelial ovarian cancer (EOC) were collected for analysis by comprehensive genomic profiling (CGP), nCounter immune profiling, and immunohistochemistry (IHQ). Biopsy samples were collected from primary lesions before EOC patients underwent carboplatin/paclitaxel-based neoadjuvant chemotherapy, according to availability. Surgery samples were extracted from residual (matched) lesions after treatment with chemotherapy, according to availability. For each technique independently, the number of patients and available material (biopsy and surgery samples) is indicated in the figure, as well as the number of samples finally included in the various analyses. The three tables summarize the total number of patients, their corresponding samples (only biopsy, only surgery, or both) available for the different analyses, and corresponding chemotherapy response scores (CRS1/2 and CRS3).

We collected a total of 60 EOC patients with sufficient viable tumor content in biopsy and/or surgery samples. The Ethical Committee for Clinical Research of “University Hospital 12 de Octubre” approved the study. Samples and clinical united data from patients were provided by the Biobanco i+12 in Hospital 12 de Octubre integrated with the Spanish Hospital Biobanks Network (RetBioH; www.redbiobancos.es), following standard operating procedures with appropriate approval from the Ethical and Scientific Committee (CEIC16-011). The patients/participants provided their written informed consent to participate in this study. A detailed overview of the ovarian cancer patient cohort and material available for this study is provided in Fig. 7. Formalin-fixed paraffin-embedded (FFPE) tumor specimens with at least 20% viable tumor content were required before the start of the study. Initial sections were stained for hematoxylin and eosin to verify the histopathological findings.

### Mutational profiling

FFPE tissue sections of histologically confirmed HGSOC or EOVC were collected and sequenced using a hybrid capture-based comprehensive genomic profiling assay (FoundationOne, Cambridge, MA, USA). This broad-based genomic panel calculates genome-wide LOH and identifies substitutions, insertions, and deletions (indels), and CNAs in codifying regions of 309 cancer genes and includes another 33 genes with selected intronic regions for the detection of gene rearrangements, *TERT*, *TERC*, and 3′UTR of *BCL2*^37^.

For the analysis of mutations, all loss-of-function alterations were considered deleterious, including deletions and frameshift or splice site alterations. For non-synonymous mutations, deleterious status was determined by manual review annotation of oncogenicity by OncoKB^38^ or recurrent mutations in Catalogue of Somatic Mutations in Cancer^39^ and consensus effect predictor indicating deleterious mutation by Varsome^40^. Gain-of-function alterations were manually reviewed in the annotation of oncogenicity by OncoKB. All high-level and focal amplifications (CN > 5) and homozygous deletion of genes known to be recurrently amplified or deleted in cancer were considered. Likely functional rearrangements (e.g. gene fusions) were considered. For mutation load, all non-synonymous protein-coding mutations identified were considered.

### Immune-related gene expression analysis

Total RNA was isolated from FFPE tissue sections using miRNeasy FFPE Kit (catalog no. 217504; Qiagen, Hilden, Germany) according to the manufacturer’s instructions and DNA was eliminated by means of Rnase-Free Dnase Set (catalog no. 79254; Qiagen, Hilden, Germany). mRNA expression was measured with the nCounter technology provided by NanoString Technologies. nCounter uses a molecular barcode technology, where the RNA is directly tagged with a target-specific capture probe and target-specific reporter probe containing a labeled barcode. Preparation and analyses were performed according to the manufacturer’s instructions using The PanCancer IO 360 gene expression panel which includes 770 immune-related genes and associated controls. Signatures were defined as previously described^41–43^. Normalization was performed by the nSolver software, correcting for the expression of technical controls and 30 housekeeping genes included in the panel. Datasets have been deposited in the Gene Expression Omnibus with accession number GSE181597. Pathway scores were used to summarize data from a pathway’s genes into a single score. Cell type scores were calculated as the average log2 normalized expression of each cell’s marker genes.

### Immunohistochemistry

Immunohistochemistry analyses were performed as previously reported^44^. Sections were blocked with 10% horse serum for 30 min, followed by incubation with the CD8 antibody (Dilution 1:10, Clone C8/144B, catalog number M7103; Agilent Dako, Santa Clara, CA, USA) diluted in 10% horse serum. Signal was amplified using avidin-peroxidase (VECTASTAIN®Elite® ABC Kit; Vector Laboraties, Burlingame, CA, USA) and peroxidase was exposed using diaminobenzidine as a substrate (DAB Substrate Kit; Vector Laboraties, Burlingame, CA, USA), according to the manufacturer’s instructions. Positive control slides were included to confirm the reactivity of the antibody. The sections were contra-stained with hematoxylin.

### Scoring systems

Immunostaining positivity was evaluated and scored. The result of immunostaining was recorded as negative or positive, taking into account the expression in both tumor and stromal areas separately. Positively stained cells only and the total number of cells were counted in five tumor and five stromal fields of each section. The percentage of positive cells in each section was calculated using the mean values.

### Statistical analyses

Comparisons of biopsy samples from patients with CRS1, CRS2, and CRS3 were performed using ordinary one-way ANOVA followed by Turkey’s multiple comparisons tests. Comparisons between biopsy and surgery samples were made with unpaired *t* test (for unpaired samples with a Gaussian distribution), Mann–Whitney test (for unpaired samples without normal distribution), paired *t* test (for paired samples showing Gaussian distribution), and Wilcoxon test (for paired samples without normal distribution). Prism 6.0 was used. *p* Value < 0.05 was considered significant. Odds ratios were calculated with Haldane correction.

### Reporting summary

Further information on research design is available in the Nature Research Reporting Summary linked to this article.

## Supplementary information

Supplementary Information

Supplementary Data 1

REPORTING SUMMARY

## Data Availability

The authors declare that all data supporting the findings of this study are available within the article and its Supplementary Information files or from the corresponding author upon request. Datasets underlying the results represented in Figs. 3, 5, and 6 and Supplementary Figs. 2, 3, 6–10 have been deposited in the Gene Expression Omnibus with accession number GSE181597. The results underlying Figs. 1, 2, and 6, Supplementary Figs. 1, 4, and 5, Supplementary Tables 1 and 2, and Supplementary Data 1 are based on a combination of genomic sequencing data from Foundation Medicine Inc. and GSK. In accordance with the Health Insurance Portability and Accountability Act, in an effort to minimize the risk of re-identification of individuals, individual-level data are not publicly available. For the Foundation Medicine dataset, raw sequencing data are proprietary and not publicly available. However, requests from accredited researchers for access to de-identified individual-level or aggregate data relevant to this manuscript, such as tumor type and mutational status, can be made available upon request by contacting L. Manso or S.W. Accredited researchers should provide contact information, affiliation/organization, and research rationale.
