# Supplementary material for: Genomic landscape and immune-related gene expression profiling of epithelial ovarian cancer after neoadjuvant chemotherapy
Source: NPJ Precis Oncol. 2022 Jan 27;6:7. doi: 10.1038/s41698-021-00247-3 (PMC8795445; doi:10.1038/s41698-021-00247-3)
Supplement: Supplementary file 1 — Supplementary Information [file 41698_2021_247_MOESM1_ESM.pdf]

**SUPPLEMENTARY INFORMATION**

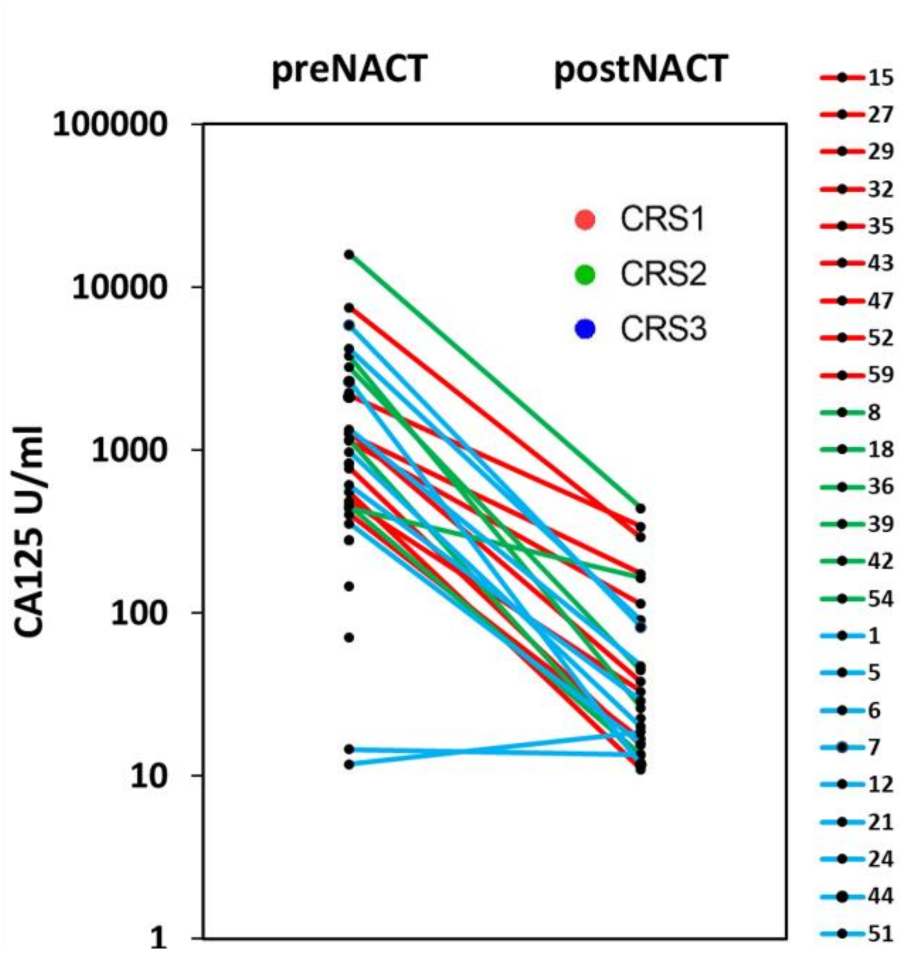


**Supplementary Figure 1. CA125 serum levels in EOC paired samples before and after NACT according to CRS patient group.** In most of the patients, CA125 biomarker levels decrease after NACT.

**Supplementary Table 1. Genomic alterations in patients subjected to NACT.**

| | All | | CRS1 | | CRS2 | | CRS3 | |
|---|---|---|---|---|---|---|---|---|
| | N° | Mean (range) | N° | Mean (range) | N° | Mean (range) | N° | Mean (range) |
| **All** | 191 | 3.75 (1-14) | 42 | 3.5 (1-9) | 88 | 4.19 (0-11) | 68 | 3.78 (0-14) |
| **Short mutations** | 85 | 1.67 (1-4) | 23 | 1.92 (1-4) | 35 | 1.67 (0-3) | 27 | 1.5 (0-2) |
| **Copy number** | 102 | 2.04 (1-13) | 18 | 1.64 (1-5) | 52 | 2.48 (0-9) | 39 | 2.17 (0-13) |
| **Rearrangements** | 4 | 0.08 (1-1) | 1 | 0.08 (1-1) | 1 | 0.05 (0-1) | 2 | 0.11 (0-1) |

Overview of all alterations found in the ovarian cancer patient cohort.

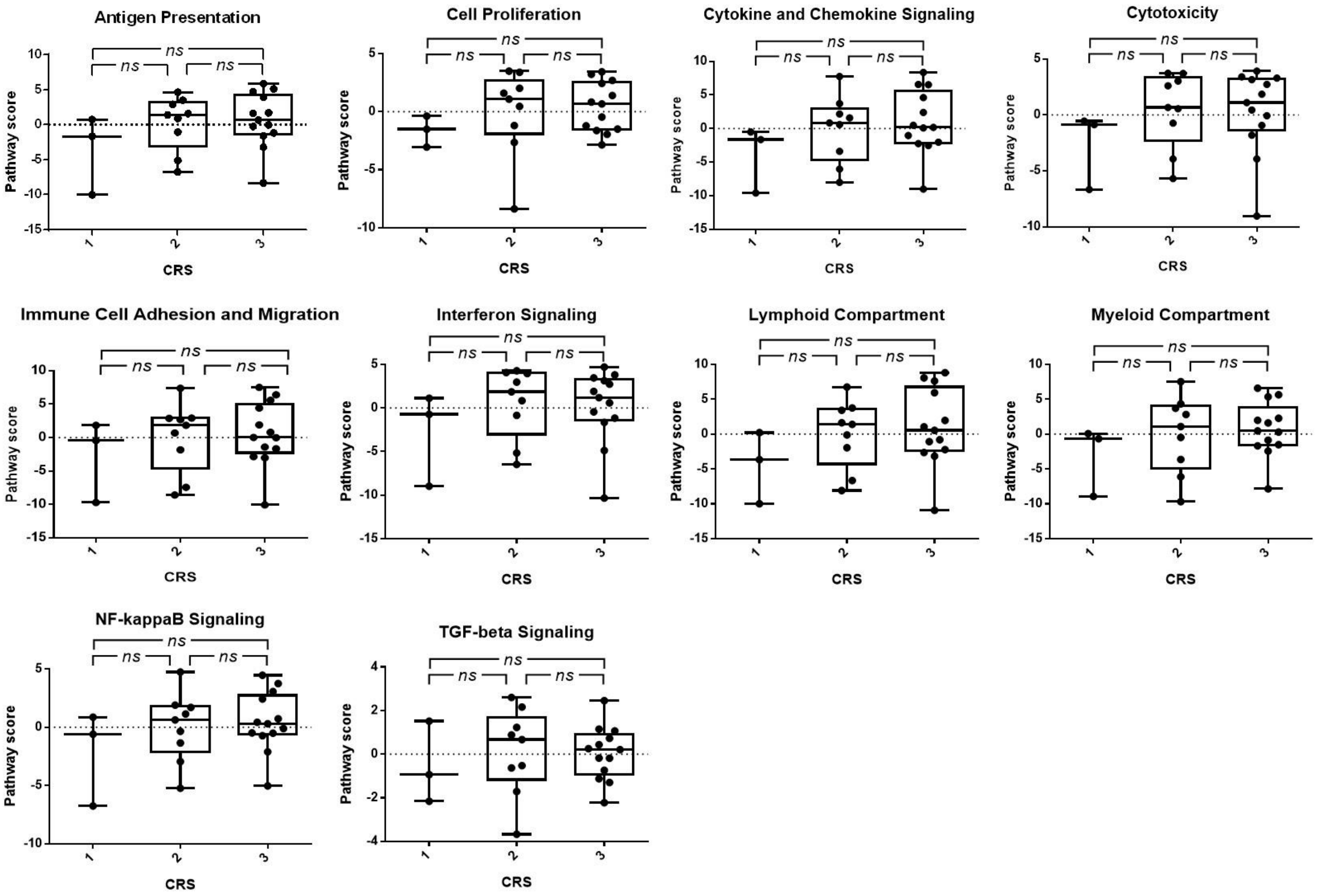


**Supplementary Figure 2. Comparison of pathway scores of pathways associated with immune regulation in EOC biopsy samples between patient groups CRS1, CRS2 and CRS3 before NACT.** No significant differences are observed in the activation of any of the pathways associated with immune regulation between EOC biopsy samples of patient groups CRS1 (n=3), CRS2 (n=9) and CRS3 (n=13) before NACT. Pathway scores were used to summarize data from a pathway's genes into a single score. The value for each EOC sample is shown. n.s. $p > 0.05$.

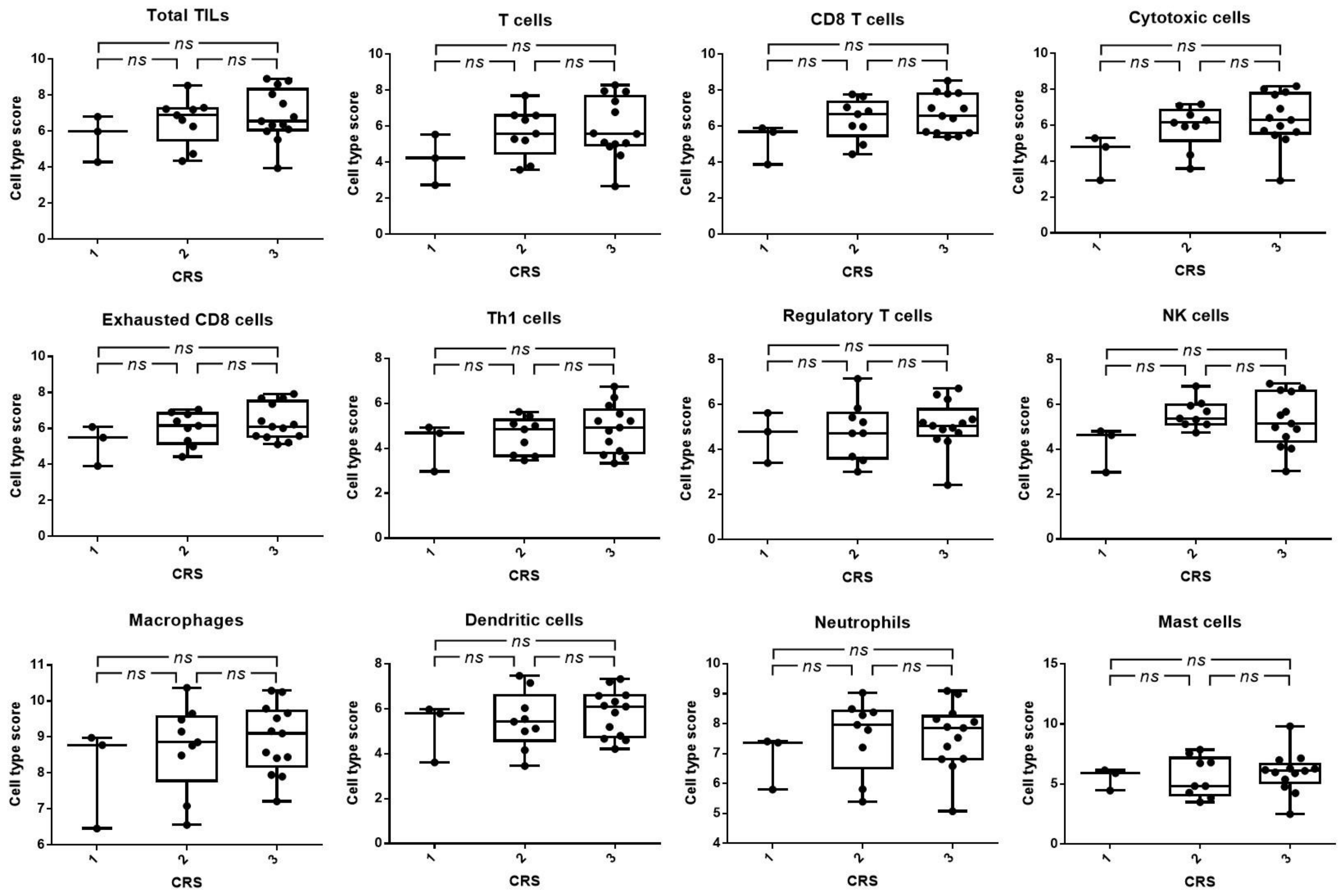


**Supplementary Figure 3. Comparison of the abundance of immune cell populations in EOC biopsy samples between patient groups CRS1, CRS2 and CRS3 before NACT.** No significant differences are found in the abundance of the different immune cell populations between EOC biopsy samples of patient groups CRS1 (n=3), CRS2 (n=9) and CRS3 (n=13) before NACT. Cell type scores were calculated as the average log2 normalized expression of each cell's marker genes. The value for each EOC sample is shown. n.s. $p > 0.05$.

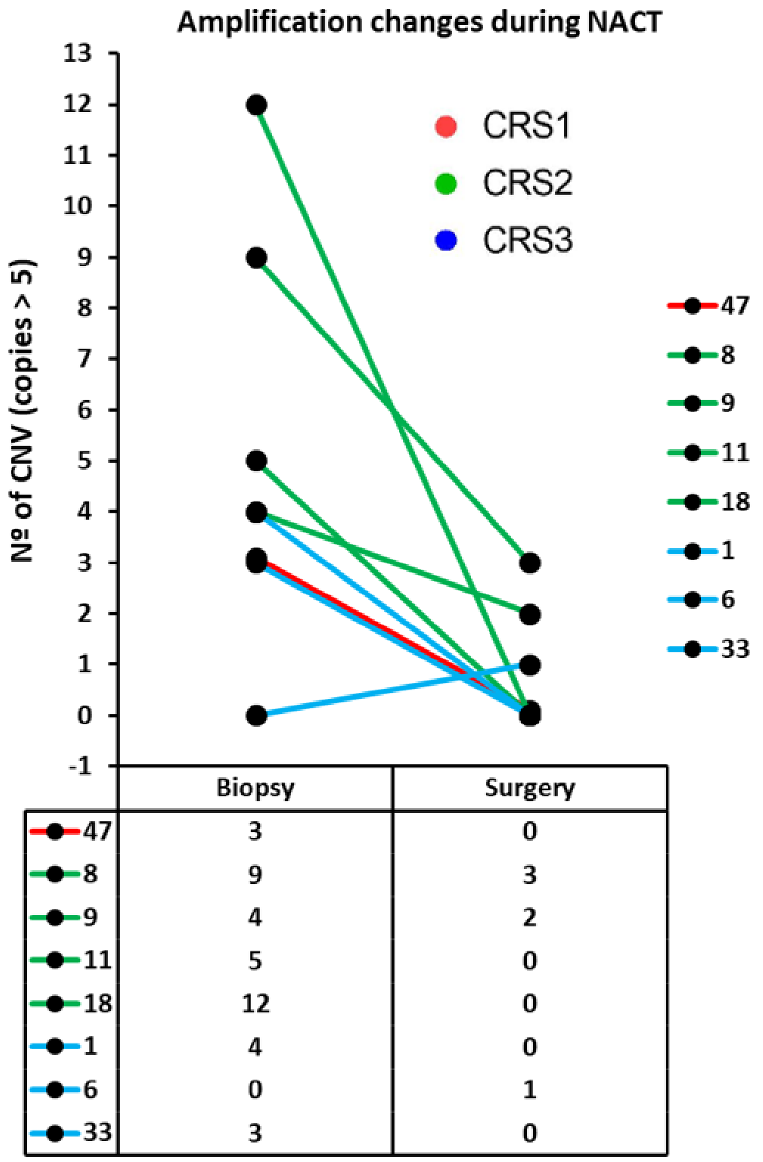


| | Biopsy | Surgery |
|---|---|---|
| 47 | 3 | 0 |
| 8 | 9 | 3 |
| 9 | 4 | 2 |
| 11 | 5 | 0 |
| 18 | 12 | 0 |
| 1 | 4 | 0 |
| 6 | 0 | 1 |
| 33 | 3 | 0 |

**Supplementary Figure 4. Amplification changes in paired samples before and after NACT according to CRS patient group.** Number of genes with amplifications (CN > 5) was quantified in each sample before and after NACT. In most of the patients, number of amplified genes decreases after NACT.

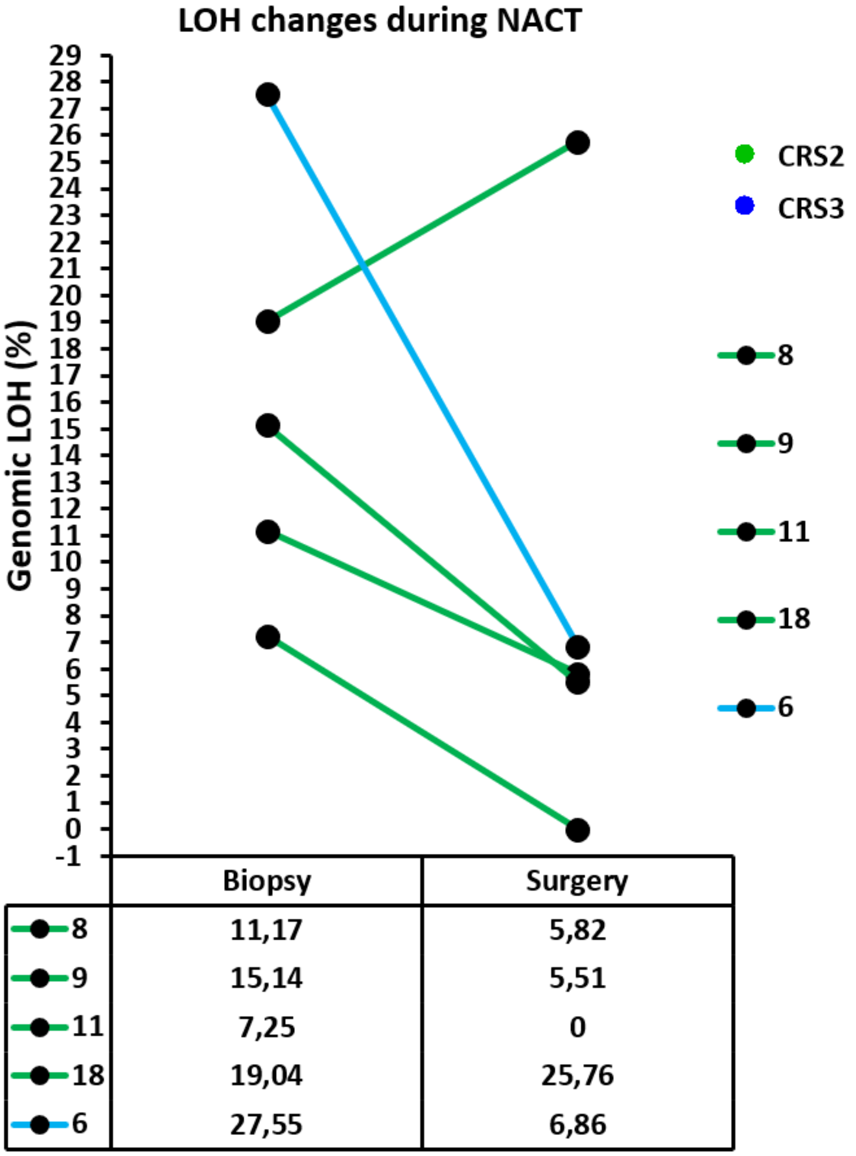


| | Biopsy | Surgery |
|---|---|---|
| 8 | 11,17 | 5,82 |
| 9 | 15,14 | 5,51 |
| 11 | 7,25 | 0 |
| 18 | 19,04 | 25,76 |
| 6 | 27,55 | 6,86 |

**Supplementary Figure 5. LOH changes in paired samples before and after NACT according to CRS patient group.** The extent of genomic LOH was quantified before and after NACT. In 4 out of 5 patients, LOH decreases after NACT.

**Supplementary Table 2. Odds ratio of pathways of responder patients (CRS3) in biopsy samples.**

| | **Odds Ratio*** | **log2 Odds Ratio** | **Tendency** |
|---|---|---|---|
| **CCNE1 vs Ras mutations** | 0.09 | -3.46 | Mutual exclusivity |
| **HRD vs PIK3 pathway** | 0.14 | -2.88 | Mutual exclusivity |
| **HRD vs Ras mutations** | 0.21 | -2.28 | Mutual exclusivity |
| **HRD vs CCNE1** | 0.52 | -0.93 | Mutual exclusivity |
| **CCNE1 vs PIK3 pathway** | 0.87 | -0.21 | Mutual exclusivity |
| **PIK3 pathway vs Ras mutations** | 1.29 | 0.36 | Co-ocurrence |

Strength of association between pathways affected by genomic alterations in EOC biopsy samples. Odds ratio was calculated with Haldane correction. “Ras mutations” include mutations in *KRAS*, *NRAS* and *NF1*; “HRD” includes mutations in *BRCA1*, *BRCA2*, *BRIP* and *ATM*; “PIK3 pathway” includes mutations in *PIK3CA*, *PTEN*, *AKT2*, *AKT3*, *PIK3C2B* and *RICTOR*; and “CCNE1” includes amplifications in cyclin E1 gene (*CCNE1*).

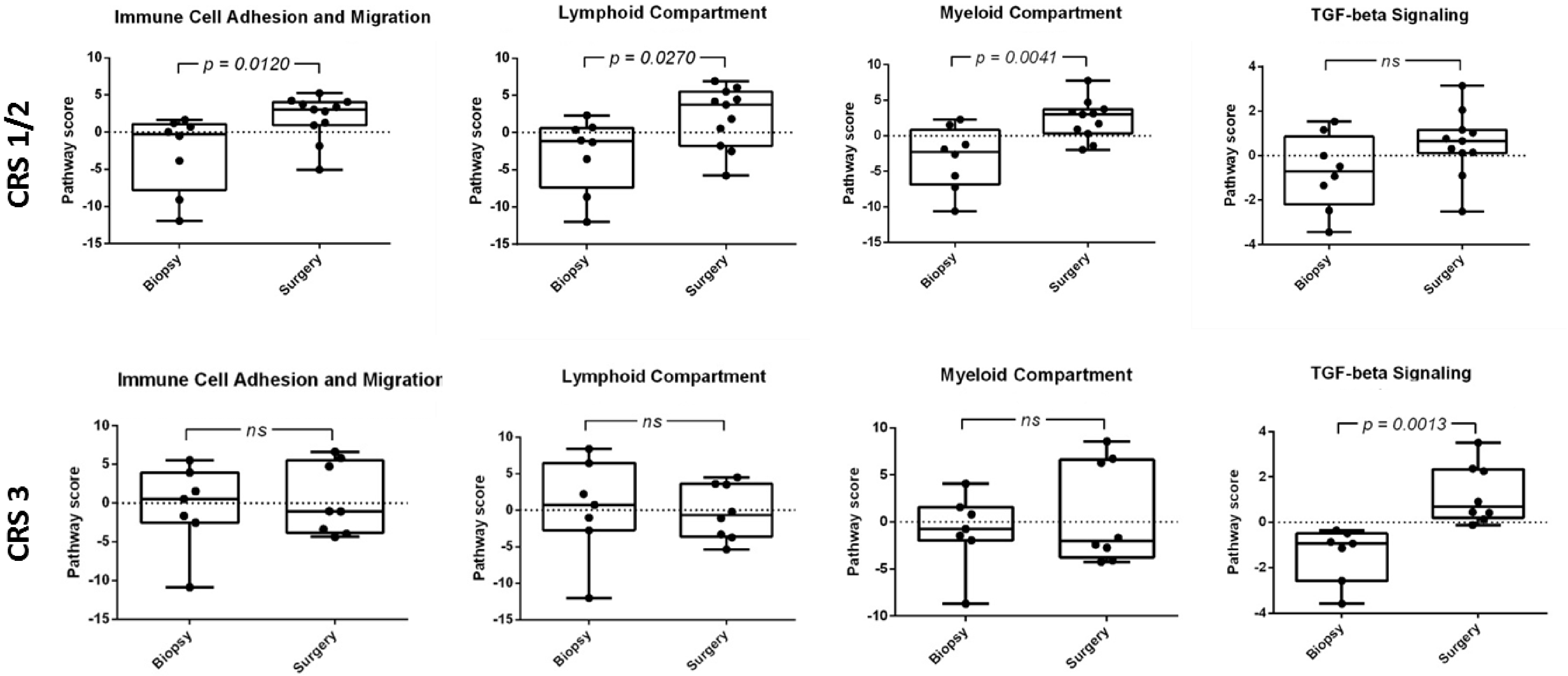


**Supplementary Figure 6. Comparison of the effect of NACT on pathways associated with immune regulation between CRS1/2 and CRS3 patients.** A significant stimulation for several pathways related to immune regulation after NACT treatment in the CRS1/2 patient population, including immune cell adhesion and migration (p=0.0120), lymphoid compartment (p=0.0270) and myeloid compartment (p=0.0041) was observed, whereas no significant stimulation of those pathways was observed in the CRS3 patient population. Immunosuppressive TGF-beta signaling was found to be significantly stimulated after NACT in CRS3 patients (p=0.0013). *P*-values were determined using the unpaired t-test. n.s. $p > 0.05$.

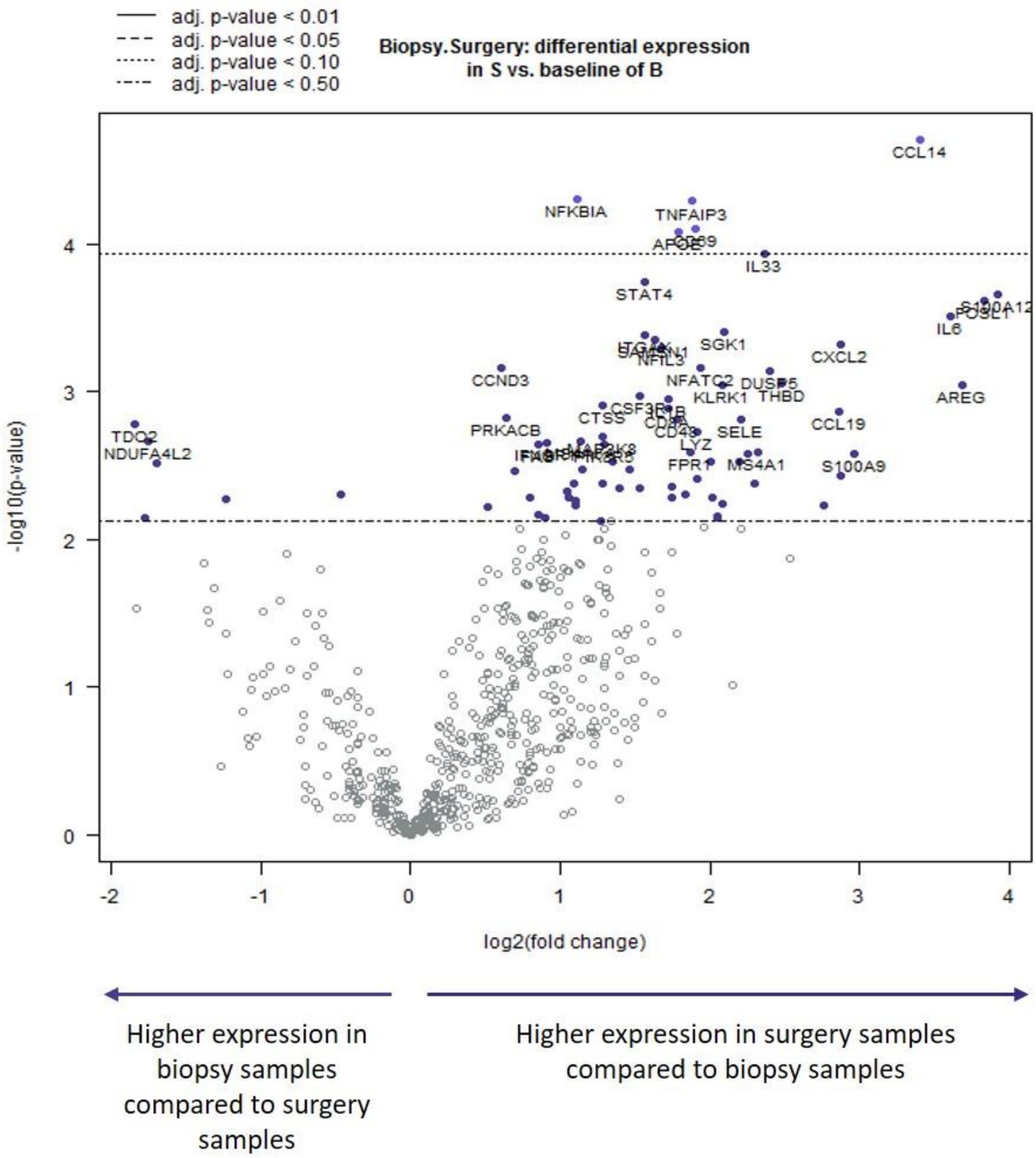


**Supplementary Figure 7. Volcano plot showing differential gene expression between biopsy and surgery samples of EOC patients (CRS1/2) treated with NACT.** Whereas the expression of many genes is increased by NACT, only a few genes show higher expression in biopsy samples compared to surgery samples.

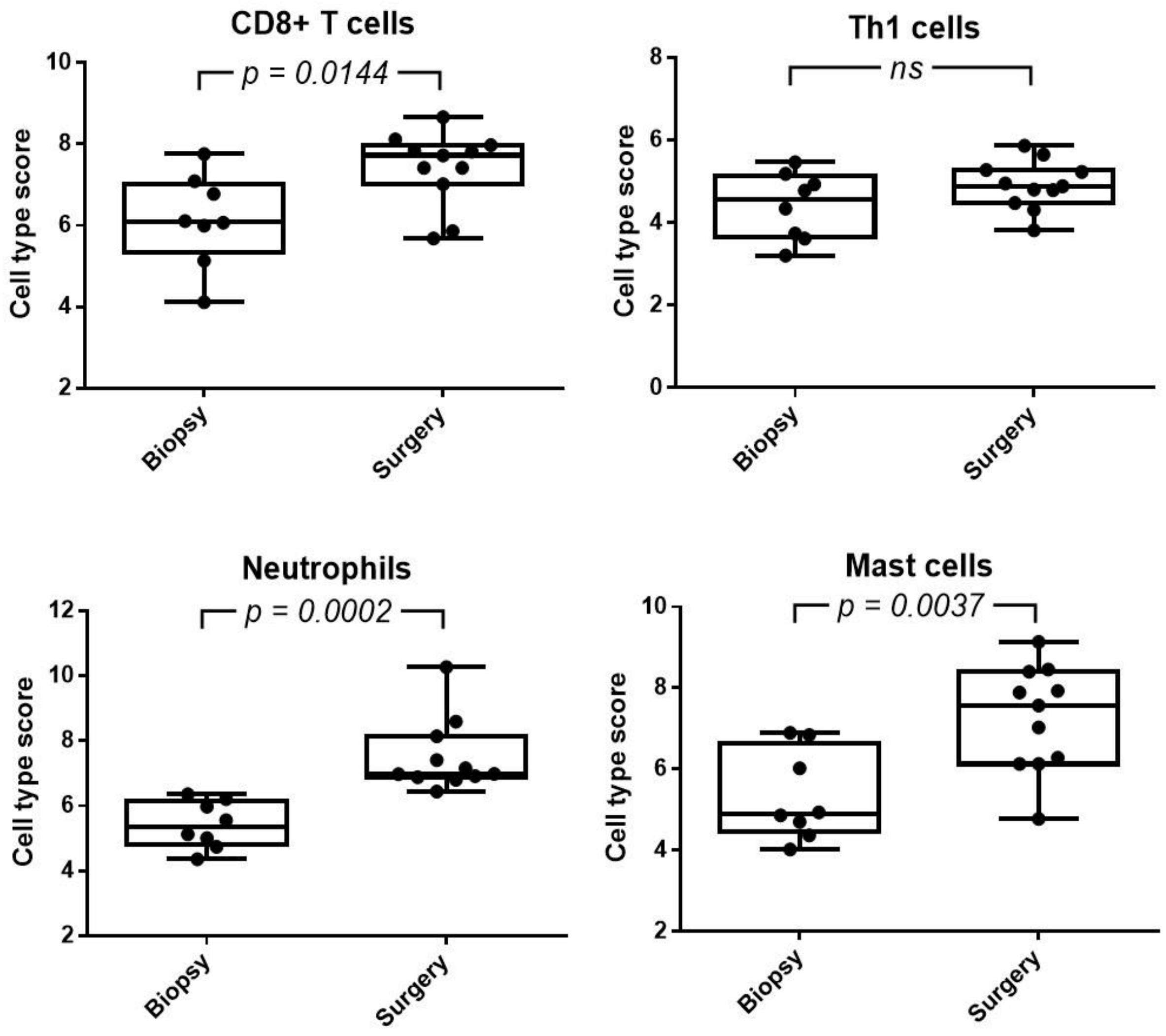


**Supplementary Figure 8. Plots representing the abundance of different immune cell subsets in EOC biopsy and surgery samples from patients with initially no/partial response to NACT.** While no difference in Th1 cell population has been found, we observe a significantly increased abundance of CD8+ T cells, Neutrophils and Mast cells in surgery samples after NACT. Cell type scores were calculated as the average log2 normalized expression of each cell's marker genes. As cell type scores are calculated in log2 scale, an increase of 1 on the vertical axis corresponds to a doubling in abundance. The value for each EOC sample is shown, biopsy samples n = 8 and surgery samples n = 11. P-values were determined using the unpaired t-test. n.s. $p > 0.05$.

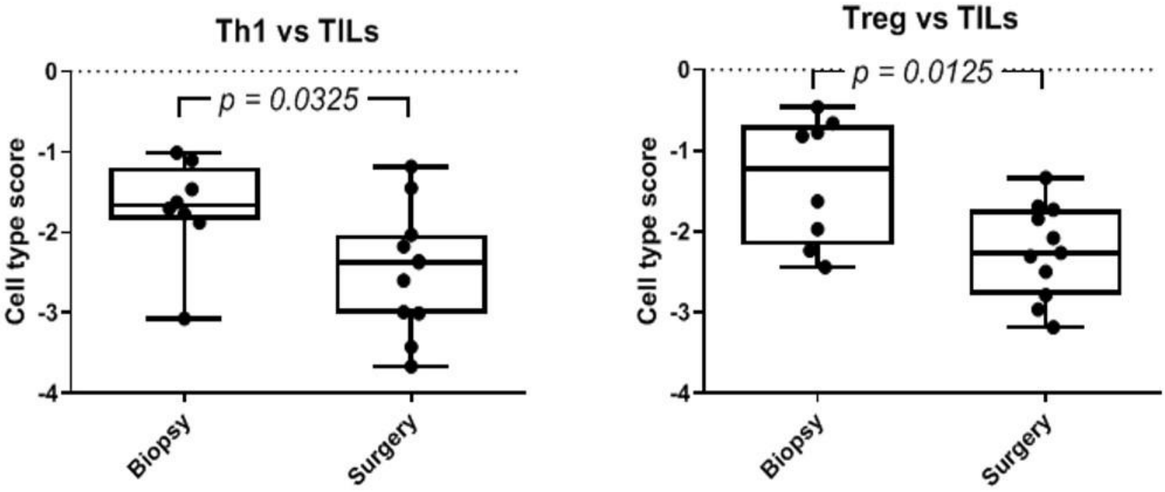


**Supplementary Figure 9. Relative abundance of Th1 vs TIL population and Treg vs TIL population in biopsy and surgery samples.** A steep decrease of Th1 cell : TIL population and Treg cell : TIL population ratios can be observed. Cell type scores were calculated as the average log2 normalized expression of each cell's marker genes. The value for each EOC sample is shown, biopsy samples n = 8 and surgery samples n = 11. *P*-values were determined using the Mann-Whitney test.

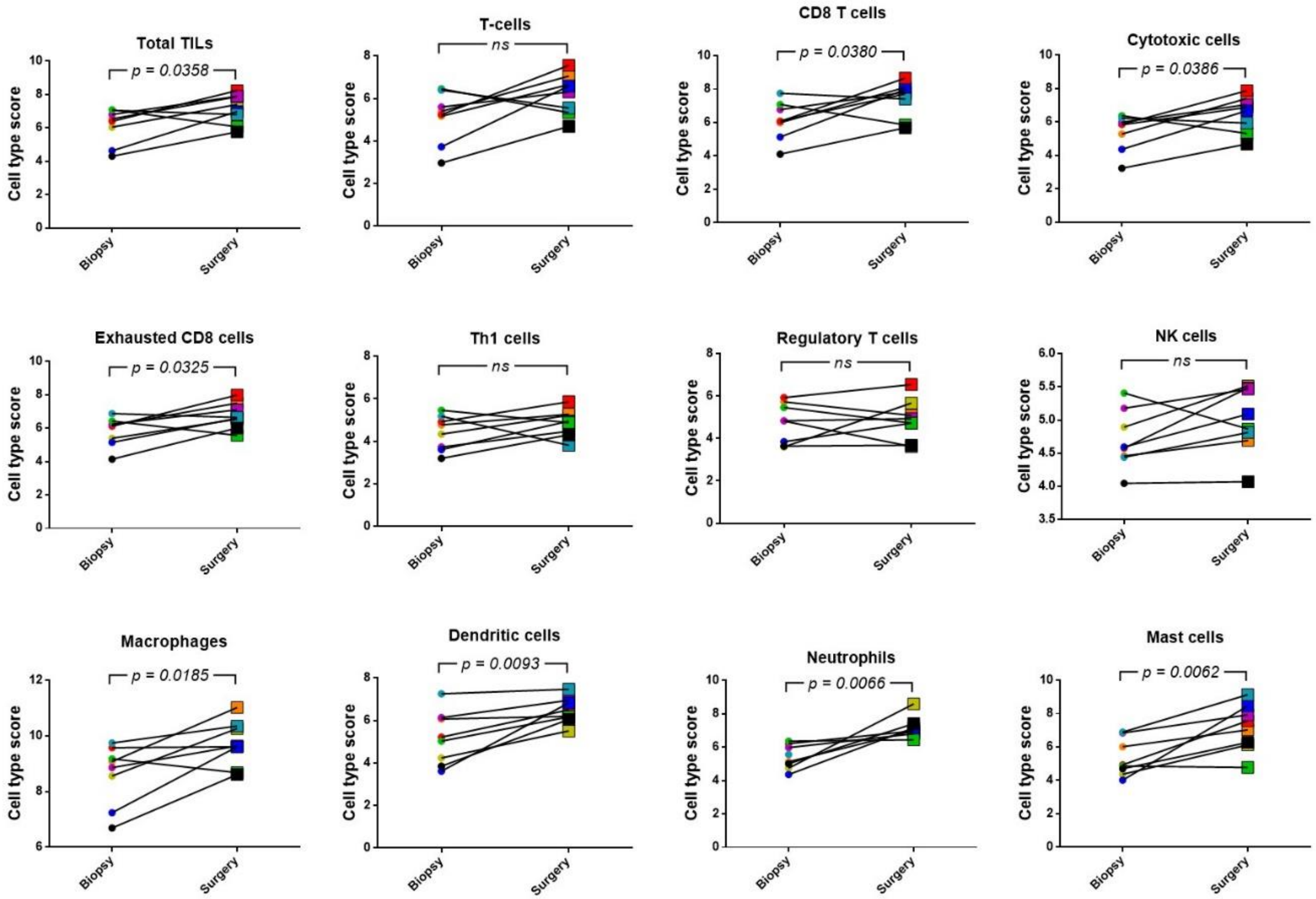


**Supplementary Figure 10. Line graphs representing the abundance of immune cell populations in paired biopsy-surgery samples of EOC patients treated with NACT.** NACT causes a significant increase in the Total TIL, CD8+ T cell, Cytotoxic cell, Exhausted CD8 cell, Macrophage, Dendritic cell, Neutrophil and Mast cell population, whereas no significant change is found in the T cell, Th1 cell, Regulatory T cell and NK cell population. Cell type scores were calculated as the average log2 normalized expression of each cell's marker genes. Each line represents an EOC patient (n=8), with colors defining the same patient in all analysis. *P*-values were determined using the paired t-test. n.s. $p > 0.05$.

**Supplementary Data 1. Comparison of genomic alterations between our EOC biopsy samples and TCGA.** Percentages of samples with one or more short-mutations, amplifications and deletions in each gene for EOC biopsy samples (n = 23) with regard to CRS and for TCGA Ovarian PanCancer Atlas (n = 585).
