# Supplementary material for: Genomic landscape and immune-related gene expression profiling of epithelial ovarian cancer after neoadjuvant chemotherapy
Source: NPJ Precis Oncol. 2022 Jan 27;6:7. doi: 10.1038/s41698-021-00247-3 (PMC8795445; doi:10.1038/s41698-021-00247-3)
Supplement: Supplementary file 3 — REPORTING SUMMARY [file 41698_2021_247_MOESM3_ESM.pdf]

nature portfolio

Corresponding author(s): NPJPRECISIONONCOLOGY-01246R1

Last updated by author(s): Nov 27, 2021

Reporting Summary

Nature Portfolio wishes to improve the reproducibility of the work that we publish. This form provides structure for consistency and transparency in reporting. For further information on Nature Portfolio policies, see our Editorial Policies and the Editorial Policy Checklist.

Statistics

For all statistical analyses, confirm that the following items are present in the figure legend, table legend, main text, or Methods section.

n/a	Confirmed	
☐	☒	The exact sample size (*n*) for each experimental group/condition, given as a discrete number and unit of measurement
☐	☒	A statement on whether measurements were taken from distinct samples or whether the same sample was measured repeatedly
☐	☒	The statistical test(s) used AND whether they are one- or two-sided<br>*Only common tests should be described solely by name; describe more complex techniques in the Methods section.*
☐	☒	A description of all covariates tested
☐	☒	A description of any assumptions or corrections, such as tests of normality and adjustment for multiple comparisons
☐	☒	A full description of the statistical parameters including central tendency (e.g. means) or other basic estimates (e.g. regression coefficient) AND variation (e.g. standard deviation) or associated estimates of uncertainty (e.g. confidence intervals)
☐	☒	For null hypothesis testing, the test statistic (e.g. *F*, *t*, *r*) with confidence intervals, effect sizes, degrees of freedom and *P* value noted<br>*Give P values as exact values whenever suitable.*
☒	☐	For Bayesian analysis, information on the choice of priors and Markov chain Monte Carlo settings
☒	☐	For hierarchical and complex designs, identification of the appropriate level for tests and full reporting of outcomes
☒	☐	Estimates of effect sizes (e.g. Cohen's *d*, Pearson's *r*), indicating how they were calculated

Our web collection on statistics for biologists contains articles on many of the points above.

Software and code

Policy information about availability of computer code

Data collection	nCounter detection software. (The PanCancer IO 360 gene expression panel) was used for data collection on immune-related gene expression, immune pathway scores as well as immune cell profiles in biopsy and surgery samples from our patient cohort
Data analysis	nSolver analysis software was used for the analysis of data on immune pathway scores as well as immune cell profiles in biopsy and surgery samples from our patient cohort.<br>Prism 6.0 was used for statistical analysis of the data obtained by nSolver advanced analysis software and for genomic data<br>Excel 365 was used for analysis of genomic data.<br>R 4.0.4 was used to process genomic data from TCGA Ovarian Pan-Cancer

For manuscripts utilizing custom algorithms or software that are central to the research but not yet described in published literature, software must be made available to editors and reviewers. We strongly encourage code deposition in a community repository (e.g. GitHub). See the Nature Portfolio guidelines for submitting code & software for further information.

Data

Policy information about availability of data

All manuscripts must include a data availability statement. This statement should provide the following information, where applicable:

- Accession codes, unique identifiers, or web links for publicly available datasets
- A description of any restrictions on data availability
- For clinical datasets or third party data, please ensure that the statement adheres to our policy

We included this statement in the manuscript: The dataset analysed during the current study are not publicly available due to potential conflict of interest but are available from the corresponding author on reasonable request.

Field-specific reporting

Please select the one below that is the best fit for your research. If you are not sure, read the appropriate sections before making your selection.

☒ Life sciences ☐ Behavioural & social sciences ☐ Ecological, evolutionary & environmental sciences

For a reference copy of the document with all sections, see nature.com/documents/nr-reporting-summary-flat.pdf

Life sciences study design

All studies must disclose on these points even when the disclosure is negative.

Sample size	From more than 600 patients with EOC entered in the Hospital 12 de Octubre Registry from 2000 to 2018, we selected a non-consecutive cohort of patients treated with NACT followed by IDS. We selected patients who: (1) had advanced high grade EOC stage III-IV according to the FIGO classification; (2) received NACT with carboplatin and paclitaxel (weekly or three-week schedule based on patient characteristics); (3) had EOC samples collected from primary lesions before patients underwent carboplatin/paclitaxel-based NACT and from residual (matched) lesions at IDS after treatment with chemotherapy; and (4) had sufficient viable tumor content (at least 20%) in biopsy and/or surgery samples. Based on these selection criteria, we started this study with a patient cohort consisting of 60 EOC patients. Our sample size has been sufficient to perform statistical analysis and to suggest new therapeutic avenues in the EOC management after NACT, but these findings will need further confirmation in larger longitudinal studies.
Data exclusions	For each technique, patients were included according to availability of material (biopsy and/or surgery sample). We didn't have enough material from all patients to be included in all techniques. For this reason, not all patients could be included in CGP analysis and nCounter gene expression analysis. After normalization and/or quality control, some samples (biopsy and surgery) were excluded before final analysis (CGP, nCounter gene expression and IHC analysis). Finally, some samples were excluded before final IHC analysis due to missing clinical data. These data have also been included in the manuscript.
Replication	Replication is not relevant to our study as we studied genomic and immune characteristics/changes by NACT using patient samples. The characteristics of each sample were analyzed once by each technique.
Randomization	Randomization is not relevant to our study as we wanted to compare genomic and immune characteristics/changes by NACT between 3 different patient groups: 1) no/minimal tumor response (CRS1), 2) partial response (CRS2) and 3) total/near-total tumor response (CRS3) to NACT. These patient groups are established following response to therapy, which was scored following guidelines recommended by the European Society for Medical Oncology (ESMO) and European Society of Gynaecological Oncology (ESGO), using the chemotherapy response score (CRS).
Blinding	Blinding was not relevant to our study as we wanted to compare genomic and immune characteristics/changes by NACT between 3 different patient groups: 1) no/minimal tumor response (CRS1), 2) partial response (CRS2) and 3) total/near-total tumor response (CRS3) to NACT. These patient groups are established following response to therapy, which was scored following guidelines recommended by the European Society for Medical Oncology (ESMO) and European Society of Gynaecological Oncology (ESGO), using the chemotherapy response score (CRS).

Reporting for specific materials, systems and methods

We require information from authors about some types of materials, experimental systems and methods used in many studies. Here, indicate whether each material, system or method listed is relevant to your study. If you are not sure if a list item applies to your research, read the appropriate section before selecting a response.

Materials & experimental systems

n/a	Involved in the study	
☐	☒	Antibodies
☒	☐	Eukaryotic cell lines
☒	☐	Palaeontology and archaeology
☒	☐	Animals and other organisms
☐	☒	Human research participants
☐	☒	Clinical data
☒	☐	Dual use research of concern

Methods

n/a	Involved in the study	
☒	☐	ChIP-seq
☒	☐	Flow cytometry
☒	☐	MRI-based neuroimaging

Antibodies

Antibodies used	CD8 antibody (Clone C8/144B, catalog no. M7103; Agilent Dako, Santa Clara, CA, USA)
Validation	https://www.agilent.com/en/product/immunohistochemistry/antibodies-controls/primary-antibodies/cd8-(concentrate)-76631#productdetails

Human research participants

Policy information about studies involving human research participants

Population characteristics	Tumor samples and medical records were analyzed from a 60 patients from Hospital "12 de Octubre" diagnosed with EOC who underwent NACT followed by IDS (see more information of selection criteria above). All patients provided written consent prior to any eligibility procedures and were enrolled in the study. Patient characteristics of this study are included in Table 1 of manuscript. Samples and data from patients included in this study were provided by the Biobanco i+12 in the Hospital "12 de Octubre" integrated in the Spanish Hospital Biobanks Network (RetBioH; www.redbiobancos.es) following standard operation procedures with appropriate approval of the RETHICAL AN Scientific Committees. Samples were collected from primary lesions before patients underwent carboplatin/paclitaxel-based NACT (so-called ´biopsy samples´), and from residual (matched) lesions at IDS after treatment with chemotherapy (so-called ´surgery samples´). All samples were FFPE tumor specimens with at least 20% tumor content.
Recruitment	No bias was generated during clinical assesment. All patients from Hospital "12 de Octubre" that fulfill the 4 criteria (see above) were included in the study
Ethics oversight	Samples and unique data from patients included in this study were provided by the Biobanco i+12 in the Hospital "12 de Octubre" integrated in the Spanish Hospital Biobanks Network (RetBioH; www.redbiobancos.es) following standard operation procedures with appropriate approval of the RETHICAL AN Scientific Committees. All patients provided written consent prior to any eligibility procedures.

Note that full information on the approval of the study protocol must also be provided in the manuscript.

Clinical data

Policy information about clinical studies

All manuscripts should comply with the ICMJE guidelines for publication of clinical research and a completed CONSORT checklist must be included with all submissions.

Clinical trial registration	*Provide the trial registration number from ClinicalTrials.gov or an equivalent agency.*
Study protocol	*Note where the full trial protocol can be accessed OR if not available, explain why.*
Data collection	*Describe the settings and locales of data collection, noting the time periods of recruitment and data collection.*
Outcomes	*Describe how you pre-defined primary and secondary outcome measures and how you assessed these measures.*
